# FoxO3a Inhibits Tamoxifen-Resistant Breast Cancer Progression by Inducing Integrin α5 Expression

**DOI:** 10.3390/cancers14010214

**Published:** 2022-01-02

**Authors:** Elena Ricci, Mariarosa Fava, Pietro Rizza, Michele Pellegrino, Daniela Bonofiglio, Ivan Casaburi, Marilena Lanzino, Cinzia Giordano, Rosalinda Bruno, Rosa Sirianni, Ines Barone, Diego Sisci, Catia Morelli

**Affiliations:** Department of Pharmacy, Health and Nutritional Sciences, University of Calabria, 87036 Rende, Italy; riccielena91@gmail.com (E.R.); mariarosafava@live.it (M.F.); pietro.rizza@unical.it (P.R.); michele.pellegrino@unical.it (M.P.); daniela.bonofiglio@unical.it (D.B.); ivan.casaburi@unical.it (I.C.); marilena.lanzino@unical.it (M.L.); cinzia.giordano@unical.it (C.G.); r.bruno@unical.it (R.B.); rsirianni@unical.it (R.S.); ines.barone@unical.it (I.B.)

**Keywords:** breast cancer, tamoxifen resistance, FoxO3a, α5-integrin, migration, invasiveness

## Abstract

**Simple Summary:**

Tamoxifen is a mainstay of adjuvant treatment for estrogen receptor α-positive (ERα+) breast tumors, which account for over 70% of all the diagnosed breast malignancies. Unfortunately, ~30% of patients initially responding to the therapy will eventually relapse with an endocrine-resistant disease. Here, we show that the transcription factor FoxO3a can overcome tamoxifen resistance by inhibiting cell motility and invasiveness, the main features of tumor progression. The underlying mechanism could be ascribed to FoxO3a-dependent transcriptional up-regulation of the integrin α5 subunit of the α5β1 fibronectin receptor, a well-known membrane heterodimer controlling cell adhesion. Indeed, FoxO3a and integrin α5 expression is significantly correlated in ERα+ tumors, and FoxO3a protective effects are lost in cells with reduced levels of integrin α5. Therefore, a pharmacological increase in FoxO3a levels could represent an effective approach to be exploited in combination with tamoxifen treatment in order to restore the sensitivity to the therapy in refractory tumors.

**Abstract:**

Resistance to endocrine therapy is still a major clinical challenge in the management of estrogen receptor α-positive (ERα+) breast cancer (BC). Here, the role of the Forkhead box class O (FoxO)3a transcription factor in tumor progression has been evaluated in tamoxifen-resistant BC cells (TamR), expressing lower levels of FoxO3a compared to sensitive ones. FoxO3a re-expression reduces TamR motility (wound-healing and transmigration assays) and invasiveness (matrigel transwell invasion assays) through the mRNA (qRT-PCR) and protein (Western blot) induction of the integrin α5 subunit of the α5β1 fibronectin receptor, a well-known membrane heterodimer controlling cell adhesion and signaling. The induction occurs through FoxO3a binding to a specific Forkhead responsive core sequence located on the integrin α5 promoter (cloning, luciferase, and ChIP assays). Moreover, FoxO3a failed to inhibit migration and invasion in integrin α5 silenced (siRNA) cells, demonstrating integrin α5 involvement in both processes. Finally, using large-scale gene expression data sets, a strong positive correlation between FoxO3a and integrin α5 in ERα+, but not in ER-negative (ERα−), BC patients emerged. Altogether, our data show how the oncosuppressor FoxO3a, by increasing the expression of its novel transcriptional target integrin α5, reverts the phenotype of endocrine-resistant BC toward a lower aggressiveness.

## 1. Introduction

Breast cancer (BC) is the most commonly diagnosed neoplasia worldwide (11.7%), even surpassing lung cancer (11.4%) [1]. Being expressed in ~70% of cases, the estrogen receptor α (ERα) is the prevalent diagnostic marker in BC. For this reason, the endocrine therapy, i.e., selective estrogen receptor modulators (SERMs as tamoxifen), selective estrogen receptor degraders (SERDs as fulvestrant), and aromatase inhibitors (AIs as anastrozole), still represents the mainstay of treatment for this subset of patients [2]. Although the therapeutic efficacy and the clinical outcomes have improved over the past three decades [3], the prolonged exposure to antiestrogen therapies may lead to the development of various adverse reactions and, more importantly, endocrine resistance, hindering the treatment effectiveness [4].

Among the molecular mechanisms that have been described to contribute to tamoxifen resistance, the overexpression of receptor tyrosine kinases (RTKs), with the consequent deregulation of downstream pathways, seems to play a crucial role [5]. In particular, the hyper-activation of the PI3K/AKT pathway has been associated with resistant phenotypes and cancer progression [6].

The Forkhead box class O3a (FoxO3a) is a member of the winged-helix transcription factors subfamily (FoxOs), whose functions are negatively regulated by the PI3K/AKT signaling [7]. In the absence of insulin and growth factors (GFs), FoxOs are mainly located within the nuclei and regulate a set of target genes involved in longevity, cell metabolism, stress resistance, cell cycle arrest, DNA damage repair, and apoptosis. In the presence of insulin or GFs, FoxOs undergo phosphorylation, bind to the chaperone proteins 14-3-3, and are retained into the cytoplasm, where they are degraded via the ubiquitin-proteasome pathway [8]. Therefore, it does not surprise that a hyperactive PI3K/AKT pathway in tamoxifen-resistant BC cells (BCCs) results in FoxO3a inactivation due to hyper-phosphorylation and subsequent degradation [9,10].

The role of FoxOs as tumor suppressor genes is well documented [11]. In cancer cells, FoxO3a is frequently inactivated by gene mutation or protein hyper-phosphorylation and consequent degradation, while FoxO3a overexpression inhibits their tumorigenic potential, proliferation, and invasiveness [12].

Moreover, FoxOs are important mediators of the functional cross-talk between GFs and estrogens, well-known players in BC development and progression [13]. Ligand-dependent ERα/FoxO3a interaction has been described [14], with FoxO3a acting as a corepressor or a coactivator for the nuclear receptor, depending on the cell system [15,16,17]. Importantly, FoxO3a seems to have a protective role in luminal-like breast cancers [9,18], but not in ERα− subtypes [19,20,21]. In this context, we previously reported that FoxO3a overexpression reduces motility, invasiveness, and anchorage-independent growth in ERα+ BC cells (BCCs) while leading to opposite effects in cells lacking ERα and that nuclear FoxO3a is inversely or directly correlated with the invasive phenotype of ERα+ or ERα− breast tumors, respectively [22].

As mentioned, FoxOs dysregulation has also been implicated in the acquisition of chemotherapy as well as endocrine resistance, mainly due to phosphorylation-driven degradation, thus inactivation, of these transcription factors by hyperactive GFs pathways in drug-resistant tumors [23,24]. In line with this assumption, we recently reported that tamoxifen-resistant BCCs do express lower levels of FoxO3a compared to parental cell lines and that its re-expression is able to restore the sensitivity to the antiestrogen and to strongly reduce tumor mass in tamoxifen-resistant mouse models [9]. Another suggested mechanism for the acquisition of a tamoxifen-resistant phenotype involves FoxO3a downregulation by miR-182-5p, being FoxO3a a miR-182-5p validated target gene. In fact, the competing endogenous RNA (ceRNA) hsa_circ_0025202, a circular RNA (circRNA) exerting antitumor activities, was found significantly downregulated in tamoxifen-resistant cells. Since hsa_circ_0025202 seems to act as a miRNA sponge for miR-182-5p, its low expression reasonably fits with higher levels of miR-182-5p and, in turn, with FoxO3a downregulation [25].

Based on this evidence, the present work wants to add new insights on the molecular mechanisms through which FoxO3a regulates tumor progression in ERα+ BCCs, with particular emphasis on tamoxifen-resistant phenotypes.

## 2. Materials and Methods

### 2.1. Cell Culture Conditions and Treatments

ERα+ human breast cancer epithelial cell lines MCF-7, ZR-75-1 (ZR-75), and T-47D, as well as MDA-MB-231 triple-negative breast cancer (TNBC), were purchased from ATCC (LGC Standards S.r.l., Milan, Italy) and authenticated as previously described [26]. MCF-7 and MDA-MB-231 cells were cultured in DMEM/F-12 medium, ZR-75 in RPMI 1640 plus 10 mM HEPES, 1 mM Na-pyruvate, and 0.24% glucose (all from Sigma-Aldrich, Merck, Milan, Italy) and T-47D in RPMI 1640 plus 0.2 U/mL insulin (Sigma-Aldrich, Merck). All media were supplemented with 10% (5% for MCF-7) fetal bovine serum (FBS), 100 IU/mL penicillin, 100 ng/mL streptomycin and 0.2 mM L-glutamine. All media and reagents were purchased from Gibco (Thermo Fisher Scientific Inc., Monza, Italy). Insulin-like growth factor-1 (IGF-1) was purchased from Sigma-Aldrich/Merck, Italy, and the PI3K inhibitor alpelisib hydrochloride was obtained from MedChemExpress (MCE, Princeton, NJ, USA).

### 2.2. Generation of Tamoxifen-Resistant Cell Lines

The selection of tamoxifen-resistant MCF-7 (TamR), ZR-75 (ZR-75/TR), and T-47D (T-47D/TR) cell lines were obtained after long-term cultivation of parental cells in their own growing medium (GM), with increasing concentrations of tamoxifen (Tam), followed by chronic exposure to Tam 1 μM, as previously reported [9]. The acquired resistance was periodically verified by checking the lack of Tam-dependent growth inhibition compared to the parental cells.

### 2.3. Generation of FoxO3a Inducible Stable Cell Lines

TamR/TetOn-AAA cell line and the relative control (TamR/TetOn-V) were developed as previously described [9], using the Tet-On Gene Expression System (Clontech, Palo Alto, CA, USA). Briefly, TamR cells were first transfected with the regulator plasmid pTet-On, carrying the geneticin (G418) resistance gene, for the selection of successfully transfected cells. G418-resistant TamR/TetOn cells were, thus, isolated and further transfected with the pTRE-F3aAAA plasmid, bearing a cDNA encoding the entire open reading frame of a constitutively active form of the human FoxO3a gene. The zeocin resistance gene in the pTRE-F3aAAA plasmid assured proper selection of double transfected cells. Control cell lines (TamR/TetOn-V) were established following the same protocol but stably transfecting the pTRE backbone (vector only) in place of the F3aAAA cDNA insert. Pools of TamR/TetOn-AAA and TamR/TetOn-V cells were collected and cultured in TamR medium containing G418 and zeocin selection antibiotics, plus Tam 1 μM. Medium was replaced every 48 h. The tetracycline derivative doxycycline (Dox) (Sigma-Aldrich, Merck, Milan, Italy) was employed to induce F3aAAA expression.

### 2.4. Plasmids and Transient Transfections

Transient expression of FoxO3a was obtained by transfecting cells, using FuGENE^®^ HD (Promega Italia S.r.l, Milan, Italy) with 1319 pcDNA3 flag FKHRL1AAA (F3aAAA), encoding the constitutively active triple mutant of FoxO3a (Addgene, plasmid 10709) or the pcDNA3.1 vector (Invitrogen, Thermo Fisher Scientific Inc., Monza, Italy) as control.

### 2.5. siRNA-Mediated RNA Interference

In order to have effective depletion of FoxO3a (siF3a) and of integrin α5 (siα5) transcripts, validated stealth RNAi (Oligo ID: VHS41092 for FoxO3a and siRNA ID: s7549 for integrin α5) were used. Cells were grown in phenol-red free-GM (PRF-GM) without antibiotics for 24 h and then silenced in suspension either with siF3a or siScramble (150 pmol/dish), using lipofectamine 2000 (Invitrogen, Thermo Fisher Scientific). Similarly, siα5 (100 pmol/60 mm dish) was employed for efficient integrin α5 knock-down. A Stealth RNAi^TM^ siRNA (siScramble) lacking identity with known gene targets was used as a negative control. All siRNA were purchased from Invitrogen, Thermo Fisher Scientific Inc. After 8 h, medium was renewed, and silencing efficiency was monitored up to 72 h.

### 2.6. Growth Assay

Cells were plated in 12-well plates (8 × 10^4^ cells/well) in PRF-GM. After 8 h, cells were synchronized overnight (ON) in PRF-serum-free medium (PRF-SFM) and then shifted in 2% PRF-charcoal-treated (PRF-CT) medium and treated or not with Tam 1 μM up to 72 h. Cells were collected, resuspended in 0.5% trypan blue solution, and counted through a Countess^®^ II Automated Cell Counter (Life Technologies, Thermo Fisher Scientific Inc., Monza, Italy).

### 2.7. Spheroid Assay

The 10^6^ cells were plated in PRF-GM in 100 mm dishes, coated with 2% agar solution in sterile PBS. Cells were monitored until spheroids formation (72 h). Images were taken at 10× magnification using an Olympus dp50 camera and ViewFinder software. Spheroids with diameters ≥ 50 μm were counted and measured using ImageJ software. Data were represented as mean ± SD of three fields per condition.

### 2.8. Colony-Forming Assay

Cells were plated (10^3^/well) in duplicate in 6-well plates in GM and treated or not with Tam 1 μM, where needed. Medium and treatment were renewed every 2 days. After 10 days, surviving colonies were fixed, stained with crystal violet, and scanned. Colonies were counted using ImageJ software 1.52t and expressed as a percentage of surviving colonies.

### 2.9. Wound-Healing Scratch Assay

Cells were plated (6 × 10^5^/well) in GM in 6-well plates. After 3 days, confluence was reached, and a scratch was made. After 24, 48, and 72 h, cells were fixed and stained with Coomassie brilliant blue. Pictures were taken at 10× magnification using phase-contrast microscopy, and the percentage of wound closure was analyzed by using ImageJ software, quantifying the wound opening area.

### 2.10. Boyden Chambers Transmigration and Invasion Assays

For migration assays, cells were detached with Versene (Gibco™, Thermo Fisher Scientific), seeded (3 × 10^4^ cells/insert) in PRF-CT on the upper face of 24-well modified Boyden chambers (8 μm) and 500 μL of PRF-GM were added to the bottom of the wells. After 16 h, migrated cells were fixed and stained with 4’, 6-diamidin-2-phenilindol (DAPI, Sigma-Aldrich, Milan, Italy), photographed (using ViewFinder 7.4.3 software connected to an Olympus dp50 camera, 10× magnification), and counted by ImageJ software.

For invasion experiments, the inner membrane of Boyden chambers was coated with 30 μL of Matrigel™ Basement Membrane Matrix (BD Biosciences, Becton Dickinson Italia S.p.A. Milan, Italy) (1:3 in PRF-SFM) and left drying at RT for 30′. The bottom of the well was filled with PRF-GM as for migration. Cells have been resuspended in PRF-CT and plated (10^5^ cells/insert) on the Matrigel layer. After 72 h, invading cells were fixed, stained, and counted as in migration experiments.

### 2.11. RNA Extraction, Reverse Transcription, and Real-Time (RT)-PCR

Total RNA was isolated using TRIzol reagent (Thermo Fisher Scientific Inc.), and 2 μg were reverse transcribed with the High-Capacity cDNA Reverse Transcription Kit (Thermo Fisher Scientific Inc.) according to the manufacturer instructions. cDNA was diluted with SYBR green Universal PCR Master Mix (Bio-Rad, Milan, Italy) and evaluated in triplicates by RT-PCR in an iCycler iQ (Bio-Rad, Milan, Italy). The following primers were used: *FOXO3* forward 5′-CAAACCCAGGGCGCTCTT-3′ and reverse 5′-CTCACTCAAGCCCATGTTGCT-3′; *ITGA5* forward 5′-GGCAGGACGCTATGTCC-3′ and reverse 5’AGGGAAGGAGGTGTGTGACTC-3′. Additional samples lacking cDNA and primers served as negative controls. Each sample was normalized on its 18S rRNA content. Relative gene expression levels were calculated on the basis of the untreated sample, and the results were reported as n-fold differences in gene expression.

### 2.12. Western Blotting (WB) Assay

Total and cytosolic proteins were extracted and quantified as previously described [22], separated on SDS-PAGE gel, and transferred to nitrocellulose membranes. Proteins were detected using the following specific monoclonal antibodies (mAbs): FoxO3a (75D8, #2497), p-FoxO3a (Ser253; #13129), integrin α5 (#4705), pAkt (Ser 473, #4060), AKT (pan) (11E7, #4685), GAPDH (14C10 #2118) (all from Cell Signaling Technology, Danvers, MA, USA), β−Actin (AC-15) (Sigma-Aldrich, Merck, Milan, Italy), Lamin B1 (Invitrogen #702972) and IRDye secondary Abs (LI-COR Biosciences GmbH, Bad Homburg, Germany). Odyssey FC Imaging System was used for image acquisitions. Protein bands were quantified by means of Image Studio™ Lite v5.2 (LI-COR Biosciences GmbH, Bad Homburg, Germany). All “Original blots and densitometries” have been included in a separate file as Supplementary Information.

### 2.13. Cloning and Luciferase Assay

The luciferase expression vector pGL3-human integrin α5 (pGL3pα5) encoding the 5′-flanking region of the human integrin α5 gene (*ITGA5*; promoter sequence NCBI accession ID: U48214), already described in a previous report from our group [27], was employed in luciferase assays. An almost canonical Forkhead responsive core sequence (FHRE) GTTTAC (nt-329/-323 from the ATG starting site) was identified using Gene Snap software on the published integrin α5 promoter sequence.

A deleted mutant (pGL3pα5-del) was obtained through PCR amplification starting from the pGL3pα5 as the template. The following primers were employed 5′–ATAAGGTACCTCTGGAAAGGAATGGGGAGG-3′ (forward); 5′–TATTCTCGAGCCC GCGCTCTTCCCTGT CC-3′ (reverse). The obtained 334 bp fragment, which lacks the Forkhead responsive core sequence (FHRE) GTTTAC (−329/−323), was digested with XhoI and KpnI and inserted into the pGL3 basic vector.

To investigate α5 transcriptional activation by FoxO3a, 7 × 10^4^ cells were seeded on 24-well plates and transfected either with pGL3pα5 or pGL3pα5-del and the Renilla reniformis luciferase expression vector pRL-Tk (Promega, USA), using FuGENE HD. Luciferase activity was assessed by means of the Dual-Luciferase^®^ Reporter Assay System (Promega, USA).

### 2.14. Chromatin Immunoprecipitation (ChIP) Assay

ChIP assay was conducted as previously described [22]. Briefly, sub-confluent cultures (65%) were cross-linked with 1% formaldehyde, lysed, and sonicated on ice for 200′’ (33 pulses of 6′’ each, with 10′’ pause after each pulse). The chromatin was immuno-cleared with salmon sperm DNA/Protein G agarose beads (Millipore, Merck, Milan, Italy) and then precipitated with anti-FoxO3a pAb (Thermo Fisher, Cat. # 720128). Immunoprecipitated DNA was analyzed by RT-PCR using a pair of primers (forward 5′-GACTCGTTTTCCGAGCGTTT-3′ and reverse 5′-GGTTAGACTGGGCGGGTTT-3′) (104 bp) mapping the FHRE core sequence (GTTTAC) on the integrin α5 promoter. Primers were designed with Snap Gene on a sequence retrieved from GeneBank Homo sapiens chromosome 12, sequence ID: NC_000012.12, length: 133275309.

### 2.15. cBioPortal Database

cBioPortal (https://www.cbioportal.org, last access 2 October 2021), a large-scale gene expression public data set from The Cancer Genome Atlas (TCGA) database, has been used to evaluate in silico the potential co-expression of *FOXO3* and *ITGA5* genes in BC patients cohorts [28]. The search was conducted over three BC mRNAs data sets: 1030 patients in “Breast Cancer (METABRIC, NATURE 2012 & NAT. COMM 2016)”, 814 patients in “Breast Cancer–Breast Invasive Carcinoma (TCGA, Firehose Legacy)”, 780 patients in “Breast Invasive Carcinoma (TCGA, Pancancer ATLAS)”. Searching criteria: tumor type (breast carcinoma) and ER status (positive or negative).

### 2.16. Kaplan–Meier Analysis

Kaplan–Meier (K–M) analysis, based on the most updated version of a public available microarray database from breast cancer patients (http://kmplot.com/analysis/index.php?p=service&cancer=breast, last access 20 December 2021), was used to evaluate the prognostic value of FoxO3a and integrin α5 in breast cancer. Distant metastasis-free survival (DMFS) and relapse-free survival (RFS) were calculated in a cohort of luminal A patients treated with tamoxifen (DMFS = 485 and RFS = 577 patients), regardless of the chemotherapy treatment. Search parameters: Gene: FoxO3a and ITGA5; Split patients by: auto-select best cutoff; Censored at threshold, selected; Use only JetSet best probe set, selected; Quality control, remove redundant samples: checked; Array quality control: exclude biased arrays selected; Restriction: luminal A; Intrinsic subtype, selected as indicated. The *p*-values were calculated using the log-rank test and plotted in R.

### 2.17. Statistical Analysis

Statistical analysis was performed using Student’s t-test and ordinary ANOVA test through the GraphPad Prism software. All data are reported as the mean ± standard deviations (s.d.) of at least three different experiments. * *p* ≤ 0.05, ** *p* ≤ 0.01, *** *p* ≤ 0.001 vs. control.

## 3. Results

### 3.1. FoxO3a Re-Expression Reduces the Aggressiveness of Tamoxifen-Resistant BCCs

**Figure 1:**
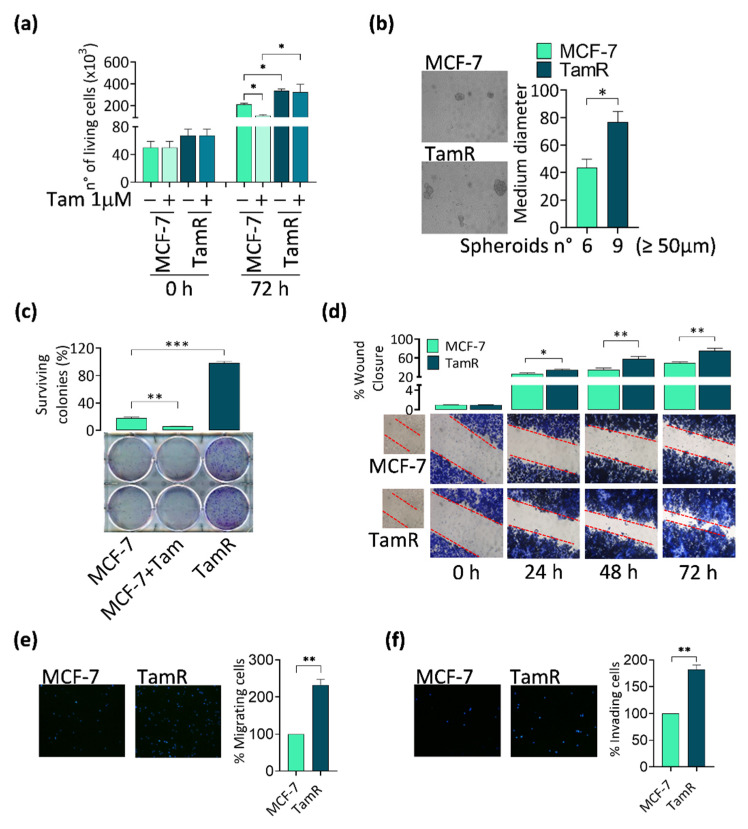
Comparison of tumor progression hallmarks in parental and TamR-derived BCCs. (**a**) Proliferation rate, (**b**) spheroids and (**c**) colony-forming potential, (**d**) wound-healing ability, as well as (**e**) motility and (**f**) invasivity have been compared in parental MCF-7 and the derived TamR cell line (refer to Section 2 for experimental procedures). Results represent the mean ± s.d. of at least three independent experiments (* *p* ≤ 0.05, ** *p* ≤ 0.01, *** *p* ≤ 0.001).

**Figure A1:**
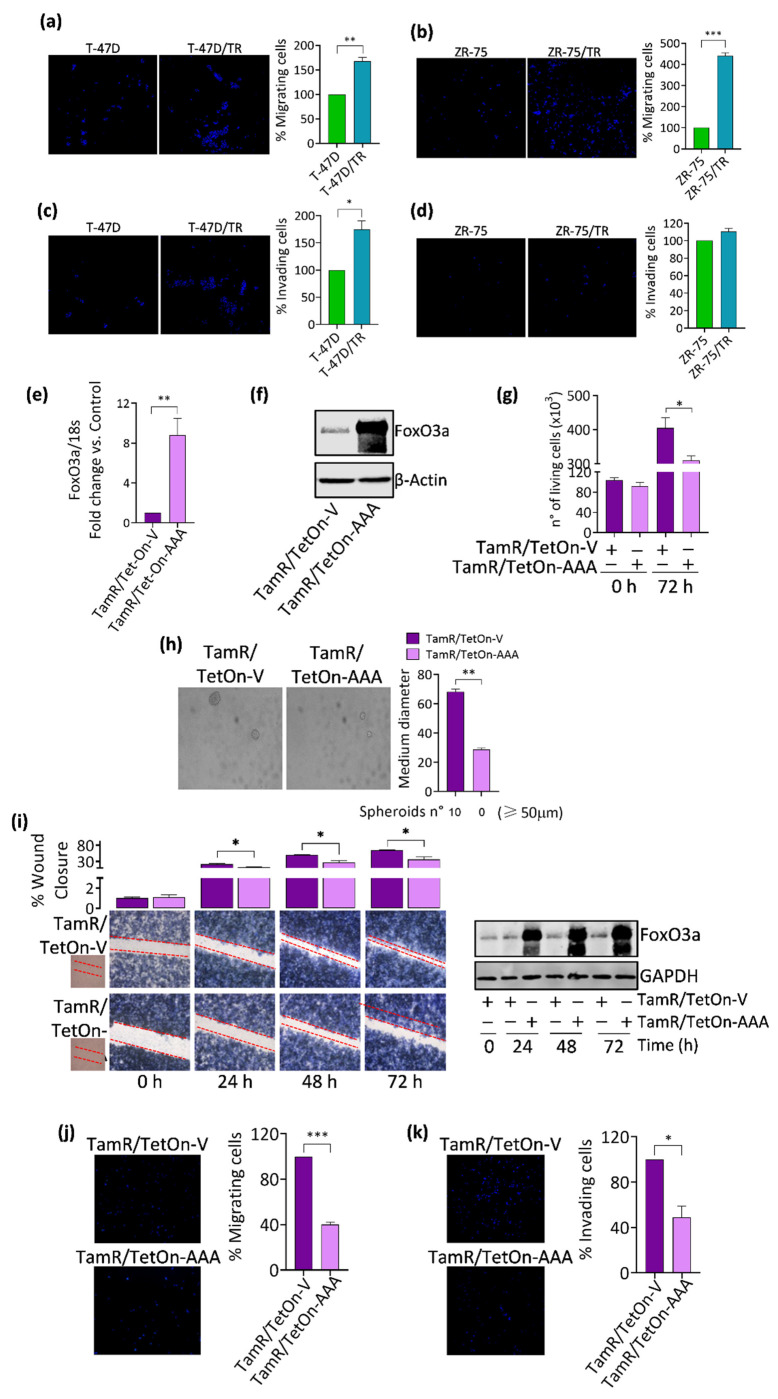
A re-expression affects the progression of TamR BCCs. Tamoxifen-resistant T-47D/TR and ZR-75/TR and the relative parental cell lines were subjected to migration (**a**,**b**) and invasion (**c**,**d**) experiments, as described in Section 2. Dox-inducible FoxO3a/TamR stable cell line (TamR/TetOn-AAA) and the corresponding control cell line (TamR/TetOn-V) were treated with Dox 1 µg/mL for 48 h. The efficiency of FoxO3a induction was evaluated both at the (**e**) mRNA and (**f**) protein levels. mRNA content was normalized vs. its 18S rRNA content. β-Actin was used as a loading control (**g**). Cells were seeded in PRF-GM plus Tam 1 μM. The day after, medium was refreshed, and cells were treated with Dox 1 µg/mL. Medium was replaced every 48 h, and cells were counted after 72 h. (**h**) Cells (10^6^) were seeded in GM on agar stratified 100 mm dishes, treated with Dox 1 µg/mL, and monitored for up to 72 h. Dox was renewed after 48 h. Images were taken at 10× magnification. The graph represents the mean of spheroids number and size. (**i**) Confluent cells were subjected to wound-healing scratch assay and treated with Dox 1 µg/mL. Dox-induced FoxO3a (TamR/TetOn-AAA) significantly hinders wound closure at all time points compared to the control cell line (TamR/TetOn-V). Proteins were collected from a duplicate set of cells and subjected to WB analysis to assess the efficiency of FoxO3a induction (right panel). Cells were seeded, treated with Dox 1 µg/mL for 48 h, and then harvested with Versene and subjected to migration (**j**) and invasion (**k**) assays. All graphs represent the mean ± s.d. of at least three independent experiments (* *p* ≤ 0.05, ** *p* ≤ 0.01, *** *p* ≤ 0.001).

The acquisition of a tamoxifen-resistant phenotype has been associated with a more aggressive behavior than that of responsive tumors, exhibiting a higher proliferation rate as well as an increased migrating and invading potential in response to activated cytokines/growth factors signaling cascades [29,30]. These features have been evaluated in our TamR BCC model and compared to the originating MCF-7 cell line [9]. As expected, TamR cells do not respond to tamoxifen treatment and grow more than the parental MCF-7, not only in adhesion (Figure 1a) but also in suspension (Figure 1b), and are able to form much more and bigger colonies starting from single cells (Figure 1c). They are also much faster in closing the scratch during wound-healing assays, reaching almost the complete sealing of the gap after 72 h (Figure 1d). Transmigration experiments (Figure 1e) confirmed that the wound closure is due to increased motility and not to the higher proliferation rate of TamR cells compared to parental MCF-7 cells. Finally, TamR cells also show an increased ability in invading an artificial basement membrane in invasion assays (Figure 1f). A similar behavior was observed in two additional tamoxifen-resistant cell lines T-47D/TR and ZR-75/TR (Appendix A, Figure A1a–d).

**Figure 2:**
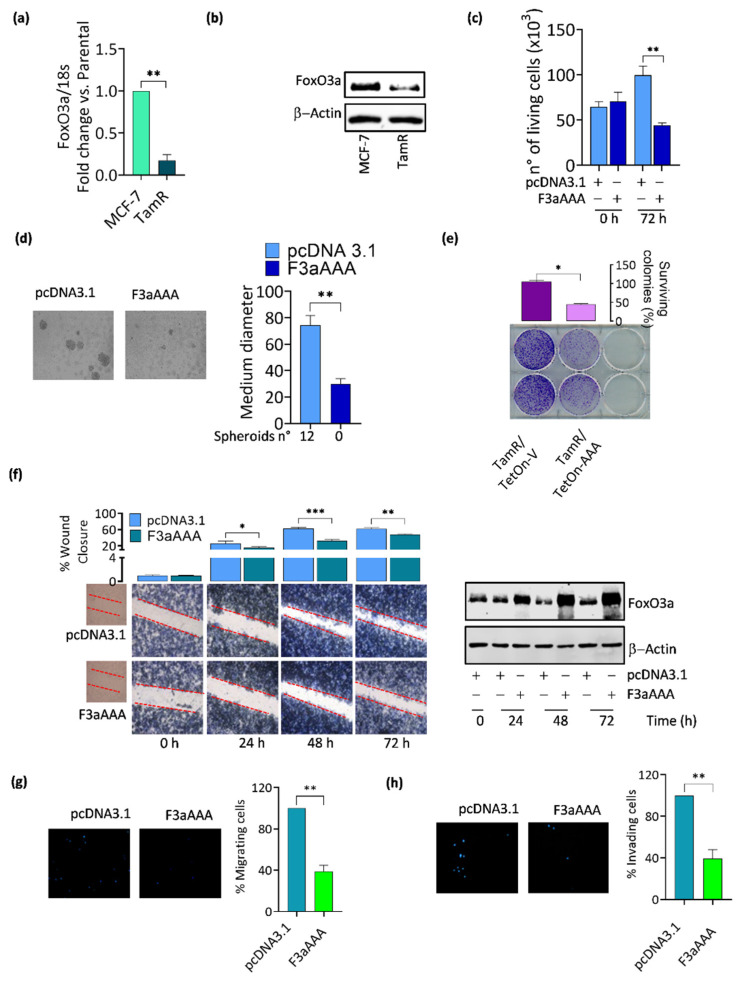
FoxO3a re-expression affects the progression of TamR BCCs. (**a**) FoxO3a transcripts were analyzed by qRT-PCR in MCF-7 and TamR cells and normalized vs. 18S rRNA content. (**b**) A total of 30 μg of cytosolic proteins were employed to evaluate FoxO3a expression in MCF-7 and TamR cells. β-Actin was used as a loading control. (**c**) TamR cells were seeded in PRF-GM plus Tam 1 µM. After 8 h, cells were starved ON in PRF-SFM. The day after, cells were switched to 2% PRF-CT and transfected either with F3aAAA or pcDNA3.1 vector as control as described in Section 2. Cells were counted after 72 h. (**d**) TamR cells were transfected in suspension with F3aAAA or pcDNA3.1, seeded on agar-coated plates, and monitored until spheroid formation (72 h). (**e**) Clonogenic assay was conducted on TamR/TetOn-V and TamR/TetOn-AAA cell lines, treated with Dox 1 μg/mL. Treatment was renewed every 48 h up to 9 days. (**f**) Confluent TamR cells were subjected to wound-healing scratch assay. Right after the scratch, cells were transfected with F3aAAA or pcDNA3.1 plasmids, and the wound closure was monitored for up to 72 h. Proteins were collected from a duplicate set of cells and subjected to WB analysis to assess FoxO3a expression (right panel). TamR cells were transfected in suspension with F3aAAA or pcDNA3.1 plasmids. After 48 h, cells were detached and seeded for migration (**g**) and for invasion (**h**) assays. Results represent the mean ± s.d. of at least three independent experiments (* *p* ≤ 0.05, ** *p* ≤ 0.01, *** *p* ≤ 0.001).

As mentioned, we previously reported that FoxO3a is significantly downregulated in TamR cells, both at the mRNA (Figure 2a) and protein (Figure 2b) level and that its re-expression is able to restore the antiproliferative response to tamoxifen in resistant cells (Figure 2c) [9]. Here, we show that FoxO3a is also a crucial modulator of processes involved in the progression of tumors that are refractory to antiestrogens. Indeed, FoxO3a overexpression in TamR cells strongly reduces their ability to grow in suspension and form spheroids (Figure 2d) as well as their clonogenic (Figure 2e), migrating (Figure 2f,g) and invading (Figure 2h) potential.

Similar results were obtained in an MCF-7-derived, Dox-inducible TamR/FoxO3a stable cell line (TamR/TetOn-AAA) when compared to the corresponding control cell line (TamR/TetOn-V), both developed as previously reported [9] and briefly described in the Section 2 section (Appendix A, Figure A1e–k). Thus, both the transient and the inducible overexpression of FoxO3a lead to comparable effects, and that no adaptive behaviors, potentially misleading, did occur in the stable cell lines. For this reason, the two experimental approaches to overexpress FoxO3a have been used indistinctly throughout the work.

### 3.2. FoxO3a Modulates the Expression of the Integrin α5 Subunit of the Fibronectin Receptor

Integrins are transmembrane receptors involved in cell adhesion to the extracellular matrix (ECM), which have been involved, both positively and negatively, in tumor initiation and progression. In this context, we and others previously reported that the integrin α5 subunit of the fibronectin receptor has a protective role in BC, behaving as an anti-metastatic factor [31], at least in ERα+ tumors [27].

**Figure 3:**
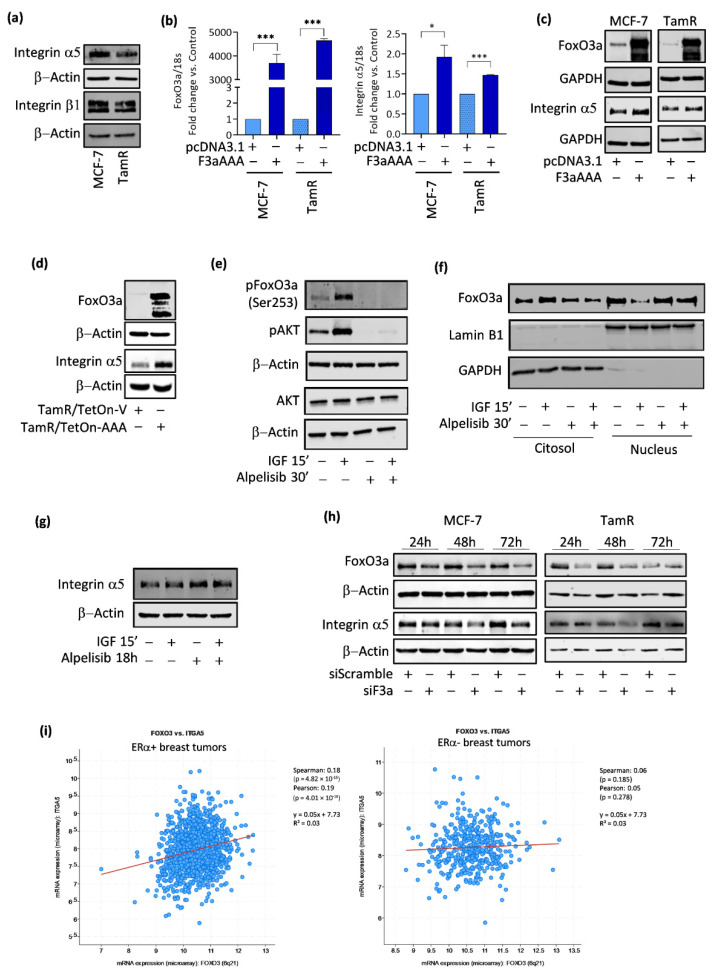
FoxO3a modulates integrin α5 expression in BC. (**a**) WB analysis was conducted on 10 μg of total lysates from TamR and MCF-7 parental cells to detect indicated proteins. β-Actin was used as a loading control. A double set of cells was transfected in suspension with F3aAAA and pcDNA3.1 plasmids for 48 h. FoxO3a and integrin α5 expression was assessed at the (**b**) RNA and (**c**) protein level. mRNA content was normalized vs. the relative 18S rRNA content, and GAPDH was employed for loading controls. (**d**) 30 μg of total proteins extracted from TamR/TetOn-AAA-derived tumors, explanted from Dox-treated mice, and from TamR/TetOn-V control tumors [9] were used to evaluate FoxO3a and integrin α5 expression. β-Actin was used as a loading control. (**e**) TamR were seeded and, the day after, they were starved for 24 h, pre-treated with alpelisib 1 μM for 30′ and then treated with IGF 10 ng/mL for 15′. Cytosolic proteins were used to detect phosphorylated proteins. β-actin was employed as a loading control. (**f**) TamR were seeded and treated as in (**e**). Cytosolic and nuclear extracts were subjected to WB analysis for the detection of indicated proteins. Lamin B1 and GAPDH were used as loading controls. (**g**) TamR were seeded, and the day after, they were starved for 8 h. Alpelisib 1 μM was added for 18 h, followed by IGF-1 treatment for 15′. Total lysates were used for integrin alpha5 detection. β-actin was used as a loading control. (**h**) WB analysis on total proteins extracted from MCF-7 and TamR cells subjected to FoxO3a silencing (siF3a). FoxO3a and integrin α5 expressions were evaluated. β-actin was served as a loading control. Results represent the mean ± s.d. of at least three independent experiments (* *p* ≤ 0.05, *** *p* ≤ 0.001). (**i**) mRNA transcripts analysis through cBioPortal large-scale was used to investigate FoxO3a (FOXO3) and integrin α5 (ITGA5) correlation in ER+ (left panel) and ER− (right panel) breast tumors. Spearman and Pearson correlation coefficients with the respective *p*-value, regression line and R2 are reported.

**Figure A2:**
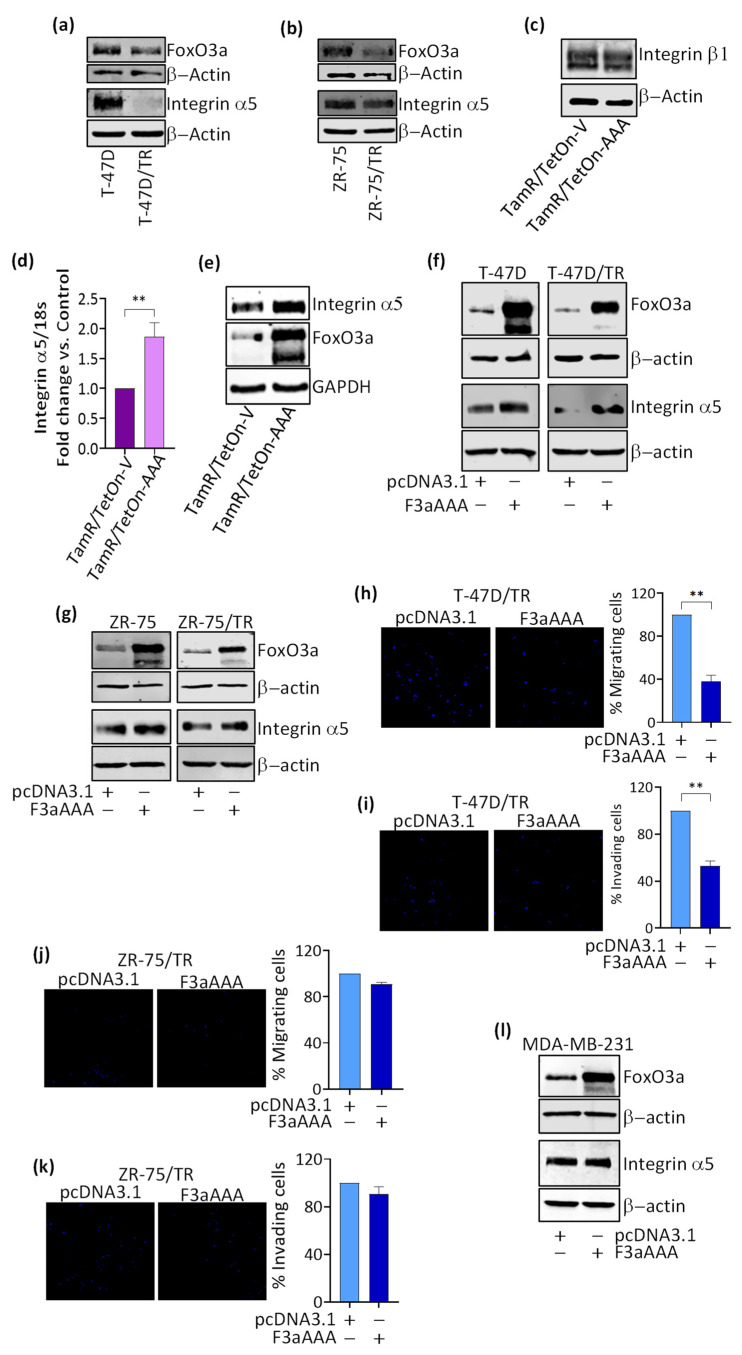
FoxO3a induces integrin α5 expression in ERα+ but not in ERα− BCCs. WB analysis of (**a**) T-47D, (**b**) ZR-75 and their tamoxifen-resistant-derived cells (T-47D/TR and ZR-75/TR, respectively), showing a much lower expression of FoxO3a and integrin α5 in resistant cell lines, compared to parental cells. (**c**) Total proteins were extracted from TamR/TetOn-AAA and from TamR/TetOn-V control-derived tumors, previously explanted from Dox-treated mice [9], and subjected to WB analysis to evaluate β1 expression (β-Actin: loading control). Dox-induced FoxO3a (for 48 h) increased integrin α5 mRNA (**d**) and protein (**e**) content in TamR/TetOn-AAA cells but not in control TamR/TetOn-V cells. A higher protein expression of integrin α5 was observed in the other two FoxO3a overexpressing, ERα+ BC cell lines, (**f**) T-47D and (**g**) ZR-75 as well as in their tamoxifen-resistant counterparts. FoxO3a ectopic expression also affected T-47D (**h**,**i**) and, in a lesser extent, ZR-75 (**j**,**k**) motility and invasiveness. All the experiments conducted on T-47D, ZR-75, and their Tam-resistant cell lines followed the same experimental procedure already described for MCF-7 and TamR cells. (**l**) FoxO3a overexpression does not induce integrin α5 in ERα− BCCs: TNBC MDA-MB-231 were transiently transfected in suspension, in PRF-GM, with 3 μg/60 mm dish of F3aAAA or pcDNA3.1. After 48 h, total lysates were collected, and FoxO3a and integrin α5 content was assessed through WB analysis. β-actin was used as a loading control. Results represent the mean ± s.d. of at least three independent experiments (** *p* ≤ 0.01).

Accordingly, integrin α5 levels, but not β1, resulted in downregulated in TamR cells (Figure 3a). This might be due to increased fibronectin-driven ubiquitination and subsequent lysosomal degradation of the α5 subunit [32] in TamR cells, which express higher levels of fibronectin [33]. Lower α5 integrin levels were also observed in two additional tamoxifen-resistant cell lines, T-47D/TR and ZR-75/TR (Appendix B, Figure A2a and b, respectively). Therefore, we questioned if integrin α5 expression might be regulated by FoxO3a, having the transcription factor a similar trend of expression in TamR cells (Figure 2b). Indeed, FoxO3a overexpression (Figure 3b,c) was able to induce integrin α5, both at the mRNA (Figure 3b) and protein (Figure 3c) level. A similar integrin α5 induction was observed in vivo in FoxO3a overexpressing TamR/TetOn-AAA-derived tumors, explanted from Dox-treated mice, compared to control TamR/TetOn-V tumors (Figure 3d) [9]. Again, no significant difference was observed in β1 levels in the same tissue extracts (Appendix B, Figure A2c).

Of note, the PI3K inhibitor alpelisib, that has been recently approved in combination with fulvestrant for locally advanced or metastatic BC, by inhibiting the phosphorylation of PI3K downstream targets AKT and FoxO3a (Figure 3e) and, consequently, preserving FoxO3a nuclear sequestration, thus activation, even under GFs (IGF-1) stimulation Figure 3f), was able to slightly increase integrin α5 protein expression as soon as after 18 h incubation Figure 3g).

Finally, FoxO3a silencing (siF3a) led to an evident downregulation of the integrin α5 subunit, both in parental and TamR cells (Figure 3h), confirming that integrin α5 expression is regulated, at least in part, by this nuclear transcription factor.

### 3.3. FoxO3a and Integrin α5 Are Positively Correlated in ERα+ BC

FoxO3a-induced up-regulation of integrin α5 is not limited to TamR cells, but it also occurs in parental MCF-7 (Figure 3b,c), in the TamR-derived Tet-inducible stable cell line (Appendix B, Figure A2d,e) and in other ERα+ BC cell lines, T-47D and ZR-75, including their Tam-resistant counterparts, T-47D/TR and ZR-75/TR cells (Appendix B, Figure A2f,g, respectively). As for TamR (Figure 2b), FoxO3a is downregulated in T-47D/TR and ZR-75/TR cell lines (Appendix B, Figure A2a,b), and its overexpression significantly reduced the motility and the invasivity of transiently transfected T-47D/TR (Appendix B, Figure A2h,i). A similar trend was observed in ZR-75/TR, but FoxO3a’s effect on migration and invasion (Appendix B, Figure A2j,k) was not significant, although consistent with the lower ability of FoxO3a in inducing integrin α5 expression in these cells (Appendix B, Figure A2g). Therefore, additional factors might be involved in the control of tumor progression in this particular cell line.

On the other side, in ERα− MDA-MB-231 BCCs, FoxO3a failed to induce the integrin α5 subunit (Appendix B, Figure A2l). This result is in line with data obtained through the analysis of breast cancer data sets, available on the www.cbioportal.org website (accessed on 2 October 2021, see Section 2 for searching criteria). The analysis was conducted on ERα+ or ERα− BC patients subgroups and revealed a strong correlation between FoxO3a and integrin α5 mRNA expression only in patients with ERα+ BC, while the correlation was lost in ERα− BC patients (Figure 3i). These results suggest that the FoxO3a-dependent induction of integrin α5 is not cell type-specific, but it represents a general mechanism occurring in ERα+ breast tumors only, but not in ERα− ones.

### 3.4. FoxO3a Binds to and Transactivates the Human Integrin alpha5 Promoter

**Figure 4:**
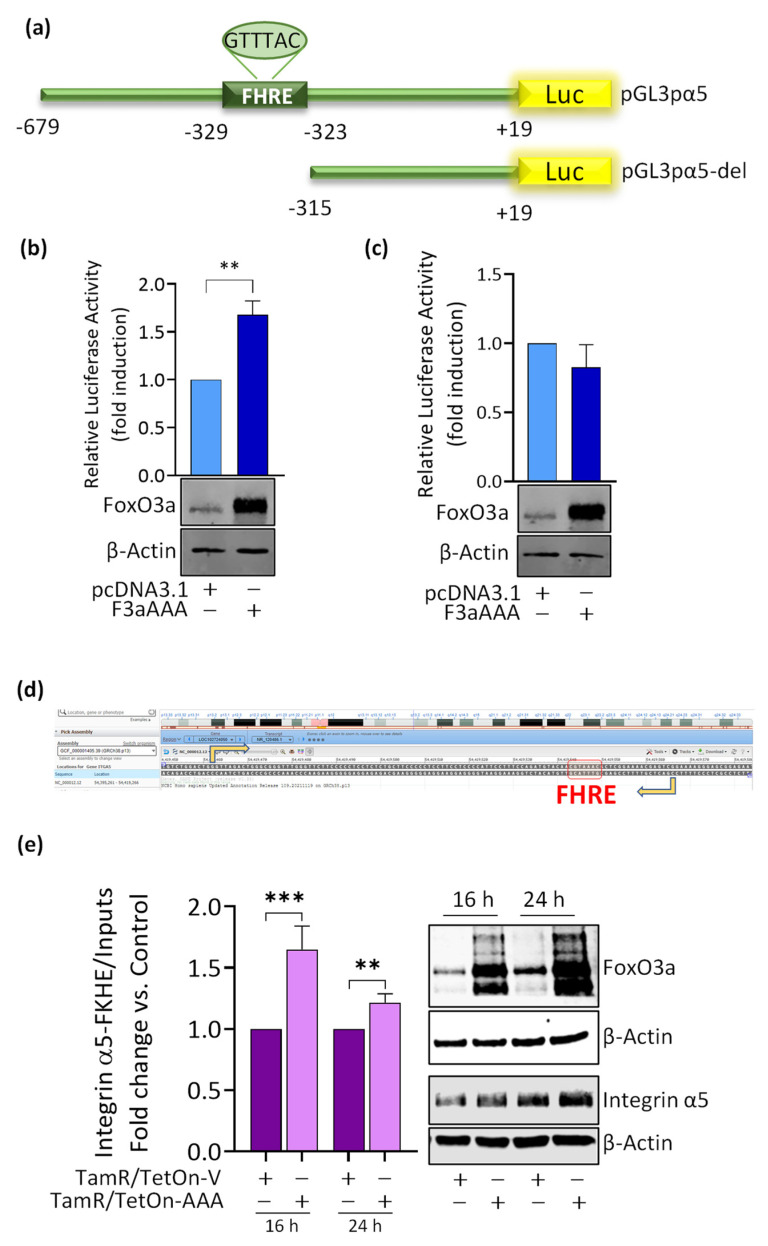
FoxO3a binds to and transactivates the human integrin α5 promoter. (**a**) Schematic representation of the human integrin α5 promoter plasmid (pGL3pα5) and the FHRE deleted fragment (pGL3pα5-del). The constructs contain the same 3′-boundary (+19), while the 5′-boundaries of the promoter were −679 and −315, respectively. (**b**,**c**) Transcriptional activity of integrin α5 promoter fragments following FoxO3a overexpression is shown. TamR cells (7 × 10^4^/well) were co-transfected in suspension either with pcDNA3.1 or F3aAAA vectors (0.5 μg/well) and seeded in GM on 24-well plates. After 36 h, a second transfection with pGL3pα5 (**b**) or pGL3pα5-del (**c**) plus pRL-Tk plasmid occurred. The next day, the firefly luciferase activity was detected. Data obtained from at least three independent experiments were normalized to the activity of the pRL-Tk and expressed as relative luciferase activity (fold induction over control). FoxO3a expression in transfected samples was analyzed by WB on total lysates from the same set of cells. (**d**) A screenshot from NCBI genome data viewer illustrating FHRE location in the 5′ flanking region of the *ITGA5* gene. FHRE motif is indicated by a red box and goes from position 54419543 to 54419550 in the negative strand. For ChIP analysis, the FHRE-containing DNA region, spanning from position 54419462 to 54419566 of the 5′flanking region of the ITGA5 gene located on chromosome 12 (**e**) was precipitated with ChIP-grade anti-FoxO3a antibody from nuclear extracts of TamR/TetOn-AAA cells, treated or not with Dox 1 μg/mL for 16 and 24 h. Purified chromatin was then amplified using a specific pair of primers (indicated by arrows in panel (**d**); see Section 2 for sequences). FoxO3a and integrin α5 expression were analyzed by WB on protein extracts from the same set of cells (right panel). Data represent the mean ± s.d. of at least three independent experiments. ** *p* ≤ 0.01, *** *p* ≤ 0.001 vs. control.

Since FoxO3a regulation of the integrin α5 occurs not only at the protein (Figure 3c) but also at the mRNA level (Figure 3b), we questioned if the nuclear factor could transcriptionally control the integrin α5 expression. Indeed, through an accurate analysis of the human integrin α5 promoter, we identified a Forkhead core sequence (FHRE), GTTTAC between the position −329/−323 from the TSS (see Section 2 for details). As supposed, FoxO3a overexpression significantly induced the activity of a vector bearing the luciferase gene under the control of the −679/+19 region of the integrin α5 promoter (pGL3pα5) (Figure 4a,b). Interestingly, the construct (pGL3pα5-del), lacking the FHRE core sequence, failed to be induced by FoxO3a (Figure 4c). The same results were obtained in TamR/TetOn-AAA cells (Appendix C).

The involvement of FoxO3a in the transcriptional activation of the α5 promoter was corroborated by chromatin immunoprecipitation (ChIP) experiments, which evidenced significant recruitment of FoxO3a on the region containing the −329/−323 FHRE sequence (Figure 4d) at 16 h and 24 h from protein induction in TamR/TetOn-AAA cells (Figure 4e). FoxO3a recruitment was consistent with the relative amounts of the transcription factor in each sample at both time points (Figure 4e, right panel). Altogether, these data suggest that the integrin α5 subunit of the fibronectin receptor is a FoxO3a-regulated gene.

### 3.5. Role of Integrin a5 in FoxO3a-Dependent Inhibition of TamR Cells Migration and Invasion and Clinical Implications

**Figure 5:**
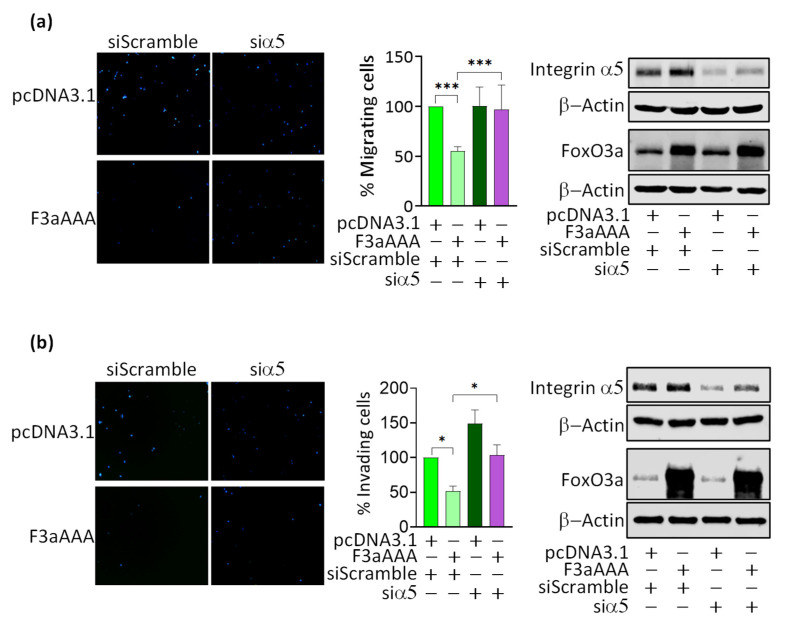

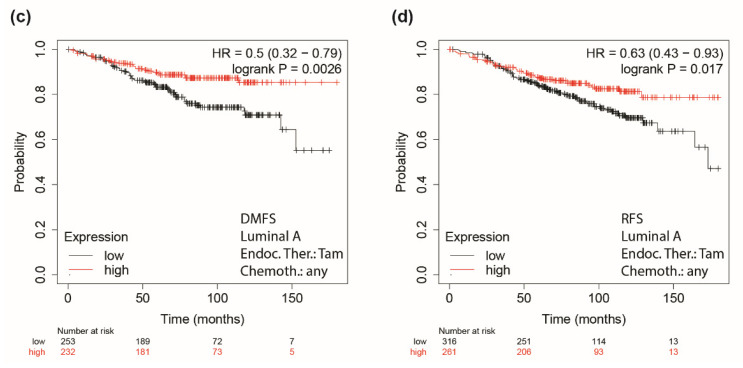
Role of integrin α5 in FoxO3a-dependent inhibition of TamR cells migration and invasion and clinical implications. TamR cells were silenced for integrin α5 as described in Section 2. The day after, cells were transiently transfected with F3aAAA for 24 h. pcDNA3.1 was used as a control vector. Cells were then employed for migration (**a**) or invasion (**b**) assays and counted using ImageJ software. The graphs report the % of migrating and invading cells. WB analysis was conducted on a duplicate set of cells to assess the effective depletion of integrin α5 and overexpression of FoxO3a. β-Actin was used as a loading control. Results represent the mean ± s.d. of at least three independent experiments (* *p* ≤ 0.05, *** *p* ≤ 0.001). Kaplan–Meier curve for (**c**) DMFS (485 patients) and (**d**) RFS (577 patients) were evaluated in a cohort of luminal A subtype BC patients treated with tamoxifen and co-expressing both FoxO3a and integrin a5 transcripts.

Finally, to assess if integrin α5 is a mediator of the inhibitory role of FoxO3a on motility and invasiveness, silencing experiments were conducted in TamR cells by using a specific and validated siRNA, targeting integrin α5 (siα5) (Figure 5). Interestingly, siα5 was able to counteract FoxO3a-dependent effects. Indeed, integrin α5 knock-down restored to control (pcDNA3.1-siScramble) levels the migrating (Figure 5a) and invading (Figure 5b) potential of F3aAAA overexpressing cells, thus confirming that integrin α5 is a mediator of the FoxO3a-dependent inhibition of TamR cells motility and invasiveness.

**Figure A4:**
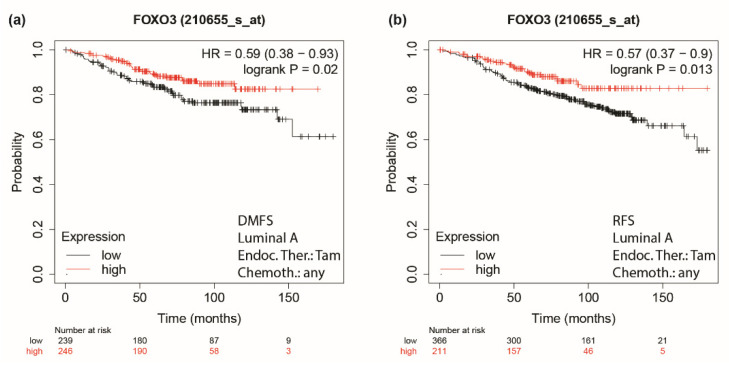
FoxOa is a positive prognostic factor in luminal A BC patients. Distant metastasis-free survival (DMFS) and relapse-free survival (RFS) were evaluated in a cohort of luminal A subtype patients treated with tamoxifen (Endoc. Ther.: Tam) (DMFS-485 patients (**a**), RFS-577 patients (**b**)). K–M analysis was performed regardless of specific chemotherapeutic treatments (Chemoth.: any). K–M are plotted for high (above median, in red) and low (below median, in black) FoxO3a gene expression. Search parameters are reported in Section 2. *p*-values were calculated using the log-rank test and plotted in R.

We previously reported how high levels of FoxO3a are associated with DMFS and RFS (see Section 2) in tamoxifen-treated luminal A BC patients only [9]. An up-to-date version of those K–M curves can be found in Appendix D, which confirms that FoxO3a correlates with a better prognosis in this subset of patients. Interestingly, in the same cohort, FoxO3a and integrin α5 co-expression strongly improve DMFS (log-rank test, *p* = 0.0026, Figure 5c) compared to FoxO3a alone (log-rank test, *p* = 0.02, Appendix D Figure A4a), corroborating the hypothesis that integrin α5 could emphasize FoxO3a protective role in ERα+ BC patients. On the other hand, FoxO3a-positive prognostic value on RFS (log-rank test, *p* = 0.013, Appendix D Figure A4b) was not significantly affected by integrin α5 co-expression (log-rank test, *p* = 0.017, Figure 5d).

**Figure 6:**
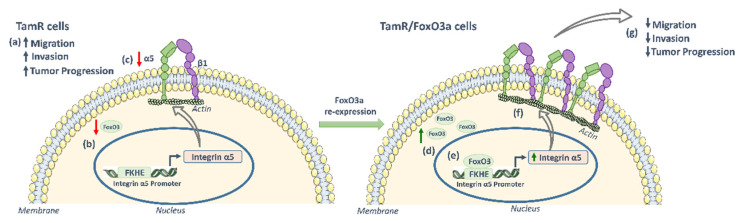
FoxO3a inhibits TamR cells progression by inducing integrin α5 expression. Compared to tamoxifen responsive MCF-7, TamR cells (a) show higher tumor progression-associated features and express reduced levels of both (b) FoxO3a and (c) integrin α5. FoxO3a re-expression (d) transactivates the human integrin α5 promoter by binding to an FHRE core sequence, (e) inducing α5 expression (f), with consequent inhibition of tumor progression (g).

A schematic representation of the main findings presented here is shown in Figure 6.

## 4. Discussion

BC is the most common female malignancy worldwide [1], with ERα positivity accounting for more than 70% of all diagnoses [34] and tamoxifen still representing the mainstay of treatment and prevention for this BC subtype [35]. A 50% reduction in the risk of relapse and a 31% reduction in the annual BC death rate was reported for 5 years of tamoxifen adjuvant therapy at 15 years of follow-up [36]. Despite this success, ~20%–30% of patients develop resistance to the treatment within 3–5 years [37]. Therefore, the search for additional molecular targets that could potentially be exploited in combination with endocrine therapies continues to be an urgent clinical need. Among the multiple mechanisms that have been described, spanning from the dysregulation of ERα signaling and cell cycle modulators to the hyper-activation of GFs pathways [38], the loss of members belonging to the Forkhead class O family has been involved in the acquisition of tamoxifen resistance. In particular, the pro-apoptotic Forkhead transcription factor FoxO1 (formerly FKHR) failed to be activated in response to tamoxifen in a TamR MCF-7 BCC model [39]. Accordingly, Vaziri-Gohar et al. observed a decreased expression of FoxO1 in MCF-7/TamR cells and reduced sensitivity to tamoxifen in FoxO1-deficient parental MCF-7 [40]. Notably, FoxO1 is a FoxO3a target gene [41]. Since we recently reported that FoxO3a is downregulated in TamR BCC models and that its induction is able to restore the sensitivity to tamoxifen both in vitro and in vivo [9], very likely that the above described FoxO1 reduction in TamR cells is a consequence of FoxO3a hyper-phosphorylation and degradation in these cells [9]. On the other hand, being the potent oncogene FoxM1, another member of the Forkhead family, negatively regulated by FoxO3a [42], its increased expression in more invasive and endocrine-resistant tumors [43,44], perfectly fits with the evidence of a defective FoxO3a in this subset of cancers.

Several other findings have demonstrated that integrins, transmembrane receptors involved in cell adhesion to ECM, are important regulators of tumor initiation and progression, cancer stemness, and drug resistance, but the available data are often conflicting [45]. For instance, subunits α6 [46] and β1 [47,48] have been positively associated with tamoxifen resistance and with tumor progression [31,49]. Nevertheless, the expression of several integrins has been found reduced in BC specimens compared with benign tumors, and it was inversely related to the presence of axillary metastasis [50]. Accordingly, the expression of integrin α5 subunit was high in non-metastatic BCCs and low in invasive cell lines, where integrin α5 overexpression was able to inhibit cell proliferation, migration, and invasion [31]. Moreover, we reported that 17β-estradiol-activated ERα increases integrin α5 expression in ERα expressing BCCs [27], suggesting that integrin α5 subunit might function as a potential metastasis suppressor, at least in ERα+ tumors.

Of note, clinical trials conducted on αVβ3/αVβ5 and α5β1 integrin inhibitors have globally failed to demonstrate therapeutic benefits in blocking tumor growth and progression, and the results were either inconsistent or even favoring placebo (!) [51].

The role of the integrin α5 subunit in antiestrogen-resistant BC has never been addressed. Here we show that, aside from FoxO3a [9], also the α5 (but not the β1) subunit of the integrin α5β1 fibronectin receptor is downregulated in TamR compared to MCF-7 parental cells. Oddly, our data are in contrast with previous observations reporting an increased β1 in a similar MCF-7-derived tamoxifen-resistant (MCF-7R) cell model, but it has to be underlined that cells were grown in quite different culture conditions [48].

We also show that FoxO3a re-expression or activation by means of PI3K/AKT pathway inhibitors induce the α5, but not the β1, integrin subunit, both in vitro and in vivo. The integrin α5 regulation likely occurs at the transcriptional level since FoxO3a is recruited to an FHRE motif identified on the *ITGA5* gene promoter. The evidence of a decreased integrin α5 expression in FoxO3a silenced cells confirmed that integrin α5 is regulated by FoxO3a.

FoxO3a-dependent increase in integrin α5 is accompanied, in turn, by a significant reduction in TamR cell migration and invasion. Interestingly, the effect was only observed in ERα+ BCCs, while integrin α5 protein levels remained unchanged in FoxO3a overexpressing ERα− cells. This evidence corroborates our previously published results, showing that ligand-activated ERα induces integrin α5 expression in ERα+ BCCs, reducing their migrating and invading potential, thus demonstrating that integrin α5 expression contributes to the maintenance of a stationary status in ERα+ cells [27]. This assumption was strongly supported by clinical data from large-scale gene expression data sets (TCGA database), which revealed a highly significant correlation between *FOXO3* and *ITGA5* expression in ERα+, but not in ERα−, BC patients (Figure 3i). Moreover, K–M analysis conducted on a tamoxifen-treated luminal A BC cohort of patients, with long-term follow-up (180 months), showed an even increased protection against recurrence and distant metastasis when both FoxO3a and integrin α5 are co-expressed (Figure 5c,d) compared to FoxO3a alone (Appendix D). Therefore, low levels of FoxO3a in association with a decrease in integrin α5 expression could identify high-risk luminal A subtype BC patients.

Altogether, our data show that the induction of integrin α5 expression might represent one of the mechanisms through which FoxO3a restores the tumor response to antiestrogen treatment, confirming that the transcription factor might represent a pursuable target to be exploited in endocrine-resistant tumors.

## 5. Conclusions

The hyper-activation of the GFs pathway is one of the most important features leading to antiestrogen resistance. Therefore, several drugs targeting the PI3K/AKT/mTOR axis are currently being investigated in clinical trials in combination with standard therapies to overcome acquired resistance to common BC treatments, including tamoxifen [6]. Despite the dysregulation of FoxOs factors has emerged as a key molecular feature of endocrine resistance mechanisms [9,24,52,53], very few clinical trials investigating the efficacy of PI3K or AKT inhibitors in BC tumors, included the evaluation of FoxO3a expression and/or subcellular localization as a secondary outcome (https://clinicaltrials.gov/, Identifiers: NCT02077569 [54], NCT02260661, NCT01339442, accessed on 10 September 2021). Among these studies, only the STAKT study, conducted on the oral selective pan-AKT inhibitor capivasertib (AZD5363), has results showing an increase in p-AKT content and FoxO3a nuclear translocation (thus activation) following treatment, which are both consistent with an inhibition of the AKT pathway [54]. However, cancer cells can employ a multitude of different mechanisms to acquire resistance to PI3K–AKT inhibition [24]. To overcome this inconvenience, restoration of FoxO3a expression and/or activity [26,55], which translates in the regulation of FoxO3a multiple transcriptional targets, including integrin α5, could represent a promising and reliable approach to be exploited as an adjuvant to antiestrogen therapy in stratified BC patients that are refractory to the treatment.

## Figures and Tables

**Figure A3:**
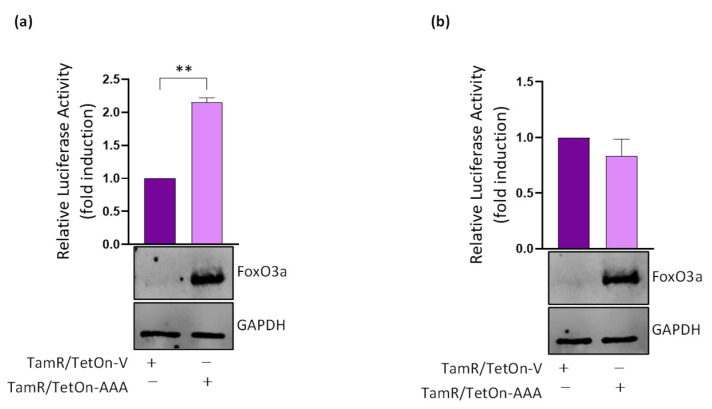
FoxO3a transactivates the human integrin α5 promoter. TamR/TetOn-AAA cells were seeded on 24-well plates in GM plus Dox 1 μg/mL. After 36 h, they were co-transfected either with (**a**) pGL3pα5 or (**b**) pGL3pα5-del and pRL-Tk. The next day cells were lysed and subjected to luciferase assay. FoxO3a expression in transfected samples was analyzed by WB on total lysates from the same set of cells. Data obtained from at least three independent experiments were normalized to the activity of the pRL-Tk and expressed as fold induction over control (** *p* ≤ 0.01).

## Data Availability

Publicly available datasets were analyzed in this study. Data can be found at: https://www.cbioportal.org and http://kmplot.com/analysis/index.php?p=service&cancer=breast.

## Supplementary Materials

The following are available online at https://www.mdpi.com/article/10.3390/cancers14010214/s1, Supplementary Information: Original blots and densitometries.

## References

[1] Sung H., Ferlay J., Siegel R.L., Laversanne M., Soerjomataram I., Jemal A., Bray F. (2021). Global Cancer Statistics 2020: GLOBOCAN Estimates of Incidence and Mortality Worldwide for 36 Cancers in 185 Countries. CA Cancer J. Clin..

[2] Shah R., O’Regan R.M. (2018). Adjuvant Endocrine Therapy. Cancer Treat. Res..

[3] Andrahennadi S., Sami A., Manna M., Pauls M., Ahmed S. (2021). Current Landscape of Targeted Therapy in Hormone Receptor-Positive and HER2-Negative Breast Cancer. Curr. Oncol..

[4] Brufsky A.M., Dickler M.N. (2018). Estrogen Receptor-Positive Breast Cancer: Exploiting Signaling Pathways Implicated in Endocrine Resistance. Oncologyst.

[5] Araki K., Miyoshi Y. (2018). Mechanism of resistance to endocrine therapy in breast cancer: The important role of PI3K/Akt/mTOR in estrogen receptor-positive, HER2-negative breast cancer. Breast Cancer.

[6] Dong C., Wu J., Chen Y., Nie J., Chen C. (2021). Activation of PI3K/AKT/mTOR Pathway Causes Drug Resistance in Breast Cancer. Front. Pharmacol..

[7] Burgering B.M., Medema R.H. (2003). Decisions on life and death: FOXO Forkhead transcription factors are in command when PKB/Akt is off duty. J. Leukoc. Biol..

[8] Calissi G., Lam E.W., Link W. (2021). Therapeutic strategies targeting FOXO transcription factors. Nat. Rev. Drug Discov..

[9] Pellegrino M., Rizza P., Dona A., Nigro A., Ricci E., Fiorillo M., Perrotta I., Lanzino M., Giordano C., Bonofiglio D. (2019). FoxO3a as a Positive Prognostic Marker and a Therapeutic Target in Tamoxifen-Resistant Breast Cancer. Cancers.

[10] Yang J.Y., Zong C.S., Xia W.Y., Yamaguchi H., Ding Q.Q., Xie X.M., Lang J.Y., Lai C.C., Chang C.J., Huang W.C. (2008). ERK promotes tumorigenesis by inhibiting FOXO3a via MDM2-mediated degradation. Nat. Cell Biol..

[11] Dansen T.B., Burgering B.M.T. (2008). Unravelling the tumor-suppressive functions of FOXO proteins. Trends Cell Biol..

[12] Liu Y., Ao X., Ding W., Ponnusamy M., Wu W., Hao X., Yu W., Wang Y., Li P., Wang J. (2018). Critical role of FOXO3a in carcinogenesis. Mol. Cancer.

[13] Lanzino M., Morelli C., Garofalo C., Panno M.L., Mauro L., Ando S., Sisci D. (2008). Interaction between Estrogen Receptor Alpha and Insulin/IGF Signaling in Breast Cancer. Curr. Cancer Drug Targets.

[14] Zou Y.Y., Tsai W.B., Cheng C.J., Hsu C., Chung Y.M., Li P.C., Lin S.H., Hu M.C.T. (2008). Forkhead box transcription factor FOXO3a suppresses estrogen-dependent breast cancer cell proliferation and tumorigenesis. Breast Cancer Res..

[15] Zhao H.H., Herrera R.E., Coronado-Heinsohn E., Yang M.C., Ludes-Meyers J.H., Seybold-Tilson K.J., Nawaz Z., Yee D., Barr F.G., Diab S.G. (2001). Forkhead homologue in rhabdomyosarcoma functions as a bifunctional nuclear receptor-interacting protein with both coactivator and corepressor functions. J. Biol. Chem..

[16] Morelli C., Lanzino M., Garofalo C., Maris P., Brunelli E., Casaburi I., Catalano S., Bruno R., Sisci D., Ando S. (2010). Akt2 Inhibition Enables the Forkhead Transcription Factor FoxO3a to Have a Repressive Role in Estrogen Receptor alpha Transcriptional Activity in Breast Cancer Cells. Mol. Cell Biol..

[17] van der Vos K.E., Coffer P.J. (2008). FOXO-binding partners: It takes two to tango. Oncogene.

[18] Habashy H.O., Rakha E.A., Aleskandarany M., Ahmed M.A., Green A.R., Ellis I.O., Powe D.G. (2011). FOXO3a nuclear localisation is associated with good prognosis in luminal-like breast cancer. Breast Cancer Res. Treat..

[19] Chen J., Gomes A.R., Monteiro L.J., Wong S.Y., Wu L.H., Ng T.T., Karadedou C.T., Millour J., Ip Y.C., Cheung Y.N. (2010). Constitutively nuclear FOXO3a localization predicts poor survival and promotes Akt phosphorylation in breast cancer. PLoS ONE.

[20] Rehman A., Kim Y., Kim H., Sim J., Ahn H., Chung M.S., Shin S.J., Jang K. (2018). FOXO3a expression is associated with lymph node metastasis and poor disease-free survival in triple-negative breast cancer. J. Clin. Pathol..

[21] Storz P., Doppler H., Copland J.A., Simpson K.J., Toker A. (2009). FOXO3a Promotes Tumor Cell Invasion through the Induction of Matrix Metalloproteinases. Mol. Cell Biol..

[22] Sisci D., Maris P., Cesario M.G., Anselmo W., Coroniti R., Trombino G.E., Romeo F., Ferraro A., Lanzino M., Aquila S. (2013). The estrogen receptor alpha is the key regulator of the bifunctional role of FoxO3a transcription factor in breast cancer motility and invasiveness. Cell Cycle.

[23] Gomes A.R., Zhao F., Lam E.W.F. (2013). Role and regulation of the forkhead transcription factors FOXO3a and FOXM1 in carcinogenesis and drug resistance. Chin. J. Cancer.

[24] Bullock M. (2016). FOXO factors and breast cancer: Outfoxing endocrine resistance. Endocr.-Relat. Cancer.

[25] Sang Y.T., Chen B., Song X.J., Li Y.M., Liang Y.R., Han D.W., Zhang N., Zhang H.W., Liu Y., Chen T. (2019). circRNA_0025202 Regulates Tamoxifen Sensitivity and Tumor Progression via Regulating the miR-182-5p/FOXO3a Axis in Breast Cancer. Mol. Ther..

[26] Pellegrino M., Rizza P., Nigro A., Ceraldi R., Ricci E., Perrotta I., Aquila S., Lanzino M., Ando S., Morelli C. (2018). FoxO3a Mediates the Inhibitory Effects of the Antiepileptic Drug Lamotrigine on Breast Cancer Growth. Mol. Cancer Res..

[27] Sisci D., Middea E., Morelli C., Lanzino M., Aquila S., Rizza P., Catalano S., Casaburi I., Maggiolini M., Ando S. (2010). 17beta-estradiol enhances alpha(5) integrin subunit gene expression through ERalpha-Sp1 interaction and reduces cell motility and invasion of ERalpha-positive breast cancer cells. Breast Cancer Res. Treat..

[28] Cerami E., Gao J.J., Dogrusoz U., Gross B.E., Sumer S.O., Aksoy B.A., Jacobsen A., Byrne C.J., Heuer M.L., Larsson E. (2012). The cBio Cancer Genomics Portal: An Open Platform for Exploring Multidimensional Cancer Genomics Data. Cancer Discov..

[29] Hiscox S., Morgan L., Barrow D., Dutkowskil C., Wakeling A., Nicholson R.I. (2004). Tamoxifen resistance in breast cancer cells is accompanied by an enhanced motile and invasive phenotype: Inhibition by gefitinib (‘Iressa’, ZD1839). Clin. Experim. Metastas..

[30] Al Saleh S., Sharaf L.H., Luqmani Y.A. (2011). Signalling pathways involved in endocrine resistance in breast cancer and associations with epithelial to mesenchymal transition. Int. J. Oncol..

[31] Wang Y., Shenouda S., Baranwal S., Rathinam R., Jain P., Bao L., Hazari S., Dash S., Alahari S.K. (2011). Integrin subunits alpha5 and alpha6 regulate cell cycle by modulating the chk1 and Rb/E2F pathways to affect breast cancer metastasis. Mol. Cancer.

[32] Lobert V.H., Brech A., Pedersen N.M., Wesche J., Oppelt A., Malerod L., Stenmark H. (2010). Ubiquitination of alpha 5 beta 1 integrin controls fibroblast migration through lysosomal degradation of fibronectin-integrin complexes. Dev. Cell.

[33] You D., Jung S.P., Jeong Y., Bae S.Y., Lee J.E., Kim S. (2017). Fibronectin expression is upregulated by PI-3K/Akt activation in tamoxifen-resistant breast cancer cells. BMB Rep..

[34] Zhang M.H., Man H.T., Zhao X.D., Dong N., Ma S.L. (2014). Estrogen receptor-positive breast cancer molecular signatures and therapeutic potentials. Biomed. Rep..

[35] Piscuoglio S., Ng C.K.Y., Weigelt B., Chandarlapaty S., Reis-Filho J.S. (2018). ESR1 and endocrine therapy resistance: More than just mutations. Ann. Oncol. Off. J. Eur. Soc. Med. Oncol..

[36] Early Breast Cancer Trialists’ Collaborative Group (2005). Effects of chemotherapy and hormonal therapy for early breast cancer on recurrence and 15-year survival: An overview of the randomised trials. Lancet.

[37] Ali S., Rasool M., Chaoudhry H., Pushparaj P.N., Jha P., Hafiz A., Mahfooz M., Abdus Sami G., Azhar Kamal M., Bashir S. (2016). Molecular mechanisms and mode of tamoxifen resistance in breast cancer. Bioinformation.

[38] Yao J., Deng K., Huang J., Zeng R., Zuo J. (2020). Progress in the Understanding of the Mechanism of Tamoxifen Resistance in Breast Cancer. Front. Pharmacol..

[39] Silva J., Cavazos D.A., Donzis E., Friedrichs W.E., Marcinialk R., Degraffenried L.A. (2007). Akt-induced tamoxifen resistance is associated with altered FKHR regulation. Cancer Investig..

[40] Vaziri-Gohar A., Zheng Y., Houston K.D. (2017). IGF-1 Receptor Modulates FoxO1-Mediated Tamoxifen Response in Breast Cancer Cells. Mol. Cancer Res..

[41] Essaghir A., Dif N., Marbehant C.Y., Coffer P.J., Demoulin J.B. (2009). The Transcription of FOXO Genes Is Stimulated by FOXO3 and Repressed by Growth Factors. J. Biol. Chem..

[42] McGovern U.B., Francis R.E., Peck B., Guest S.K., Wang J., Myatt S.S., Krol J., Kwok J.M.M., Polychronis A., Coombes R.C. (2009). Gefitinib (Iressa) represses FOXM1 expression via FOXO3a in breast cancer. Mol. Cancer.

[43] Bergamaschi A., Madak-Erdogan Z., Kim Y.J., Choi Y.L., Lu H.L., Katzenellenbogen B.S. (2014). The forkhead transcription factor FOXM1 promotes endocrine resistance and invasiveness in estrogen receptor-positive breast cancer by expansion of stem-like cancer cells. Breast Cancer Res..

[44] Millour J., Constantinidou D., Stavropoulou A.V., Wilson M.S.C., Myatt S.S., Kwok J.M.M., Sivanandan K., Coombes R.C., Medema R.H., Hartman J. (2010). FOXM1 is a transcriptional target of ER alpha and has a critical role in breast cancer endocrine sensitivity and resistance. Oncogene.

[45] Seguin L., Desgrosellier J.S., Weis S.M., Cheresh D.A. (2015). Integrins and cancer: Regulators of cancer stemness, metastasis, and drug resistance. Trends Cell Biol..

[46] Campbell P.S., Mavingire N., Khan S., Rowland L.K., Wooten J.V., Opoku-Agyeman A., Guevara A., Soto U., Cavalli F., Loaiza-Perez A.I. (2018). AhR ligand aminoflavone suppresses alpha6-integrin-Src-Akt signaling to attenuate tamoxifen resistance in breast cancer cells. J. Cell. Physiol..

[47] Pontiggia O., Sampayo R., Raffo D., Motter A., Xu R., Bissell M.J., Joffe E.B., Simian M. (2012). The tumor microenvironment modulates tamoxifen resistance in breast cancer: A role for soluble stromal factors and fibronectin through beta1 integrin. Breast Cancer Res. Treat..

[48] Yuan J., Liu M., Yang L., Tu G., Zhu Q., Chen M., Cheng H., Luo H., Fu W., Li Z. (2015). Acquisition of epithelial-mesenchymal transition phenotype in the tamoxifen-resistant breast cancer cell: A new role for G protein-coupled estrogen receptor in mediating tamoxifen resistance through cancer-associated fibroblast-derived fibronectin and beta1-integrin signaling pathway in tumor cells. Breast Cancer Res..

[49] Barnawi R., Al-Khaldi S., Colak D., Tulbah A., Al-Tweigeri T., Fallatah M., Monies D., Ghebeh H., Al-Alwan M. (2019). beta 1 Integrin is essential for fascin-mediated breast cancer stem cell function and disease progression. Int. J. Cancer.

[50] Gui G.P., Wells C.A., Browne P.D., Yeomans P., Jordan S., Puddefoot J.R., Vinson G.P., Carpenter R. (1995). Integrin expression in primary breast cancer and its relation to axillary nodal status. Surgery.

[51] Alday-Parejo B., Stupp R., Ruegg C. (2019). Are Integrins Still Practicable Targets for Anti-Cancer Therapy?. Cancers.

[52] Zhao F., Lam E.W. (2012). Role of the forkhead transcription factor FOXO-FOXM1 axis in cancer and drug resistance. Front. Med..

[53] Kumar S., Kushwaha P.P., Gupta S. (2019). Emerging targets in cancer drug resistance. Cancer Drug Resist..

[54] Robertson J.F.R., Coleman R.E., Cheung K.L., Evans A., Holcombe C., Skene A., Rea D., Ahmed S., Jahan A., Horgan K. (2020). Proliferation and AKT Activity Biomarker Analyses after Capivasertib (AZD5363) Treatment of Patients with ER(+) Invasive Breast Cancer (STAKT). Clin. Cancer Res. Off. J. Am. Assoc. Cancer Res..

[55] Taylor S., Lam M., Pararasa C., Brown J.E., Carmichael A.R., Griffiths H.R. (2015). Evaluating the evidence for targeting FOXO3a in breast cancer: A systematic review. Cancer Cell Int..

