# Supplementary material for: FoxO3a Inhibits Tamoxifen-Resistant Breast Cancer Progression by Inducing Integrin α5 Expression
Source: Cancers (Basel). 2022 Jan 2;14(1):214. doi: 10.3390/cancers14010214 (PMC8750403; doi:10.3390/cancers14010214)
Supplement: Supplementary file 1 [file cancers-14-00214-s001.zip › cancers-1489632-supplementary.pdf]

Figure 2 (b)

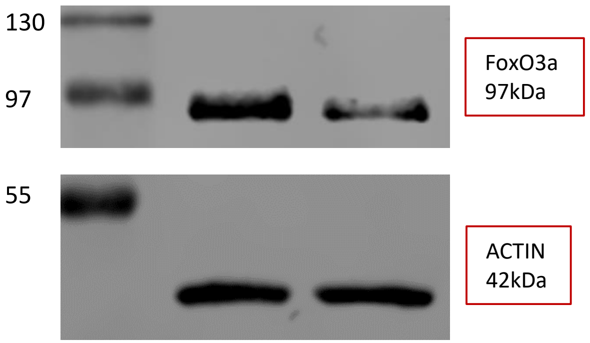

Lane	1	2
FoxO3a	14.1	7.9
Actin	26.9	27.8
Ratio	0.52	0.28
Normalized ratio	1	0.5

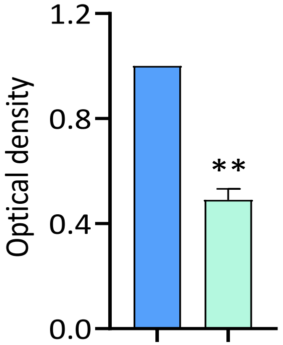

Figure 2 (f)

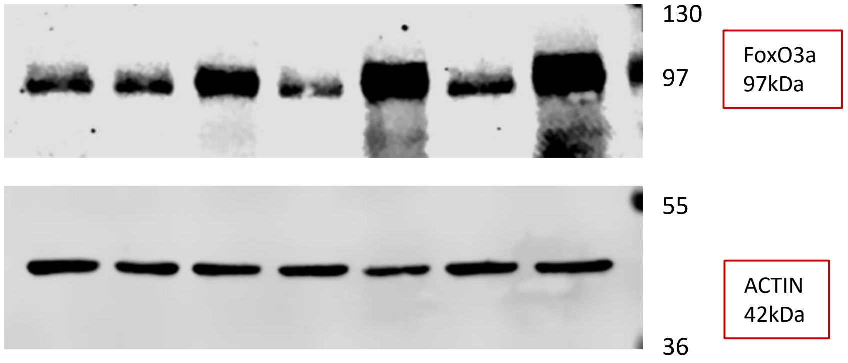

Lane	1	2	3	4	5	6	7
FoxO3a	5.6	5	15.4	4	34	6	48
Actin	23.9	19.7	16.5	15.9	10.2	19.6	20.9
Ratio	0.23	0.3	0.9	0.3	3.3	0.3	2.3
Normalized ratio	1	1.3	3.9	1.3	14.3	1.3	10

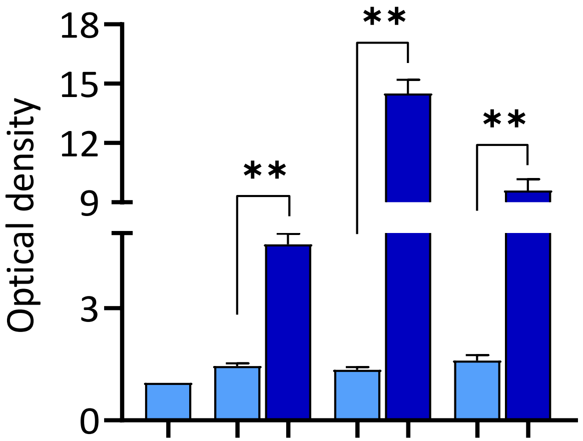

Figure 3 (a)

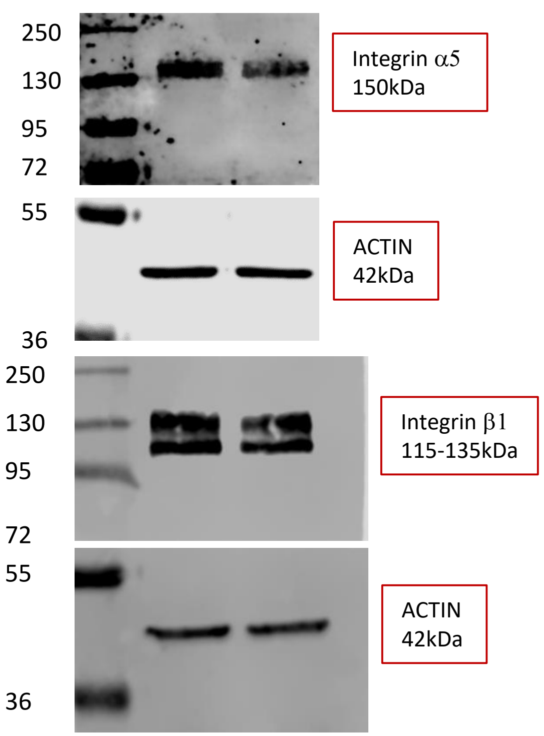

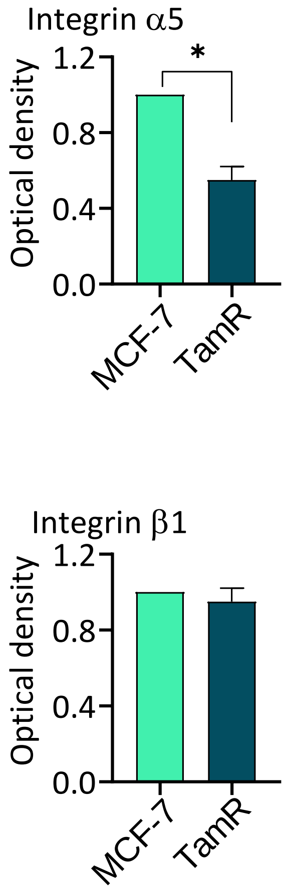

Lane	1	2
Integrin α5	4	2.5
Actin	42	38.7
Ratio	0.1	0.06
Normalized ratio	1	0.6

Lane	1	2
Integrin α5	8.49	8
Actin	18.9	18.6
Ratio	0.45	0.43
Normalized ratio	1	0.96

Figure 3 (c)

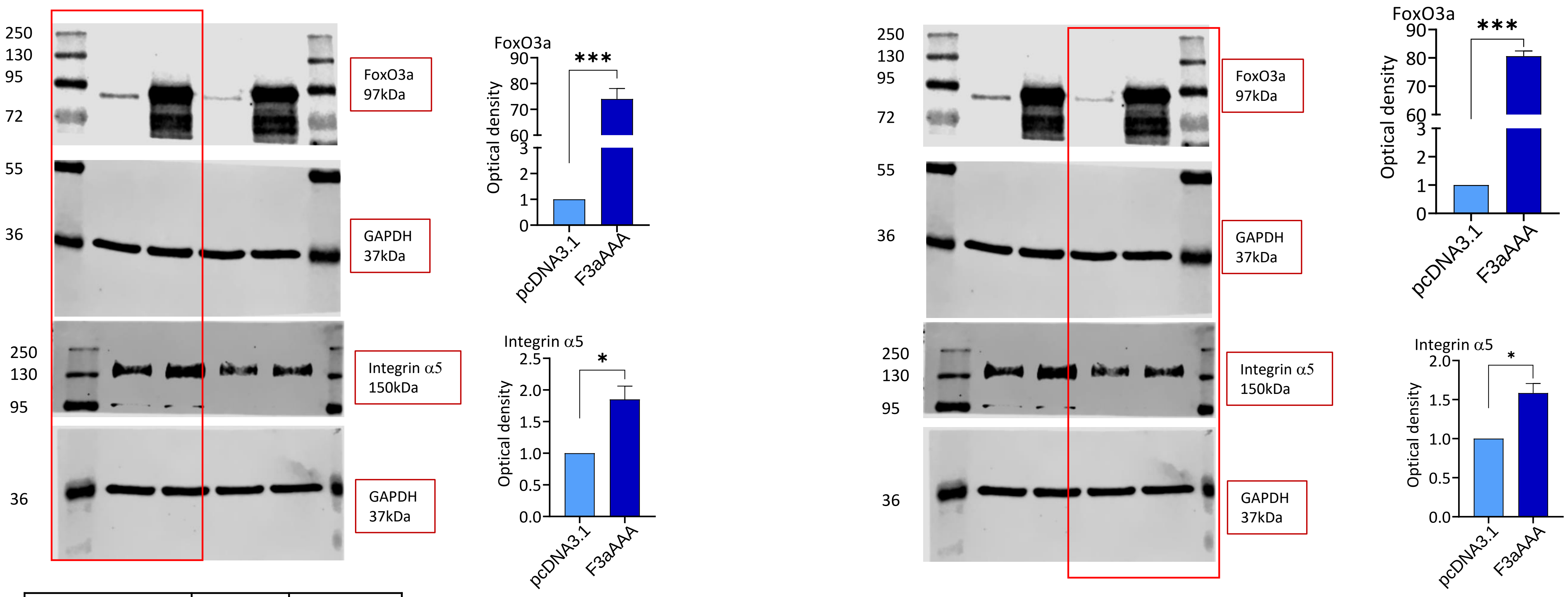

Lane	1	2
FoxO3a	4	280
GAPDH	40.5	39.4
Ratio	0.1	7.1
Normalized ratio	1	71

Lane	1	2
Integrin α5	5.3	7.6
GAPDH	40.1	38.3
Ratio	0.1	0.2
Normalized ratio	1	2

Lane	1	2
FoxO3a	1	105
GAPDH	39	42.3
Ratio	0.03	4.5
Normalized ratio	1	83

Lane	1	2
Integrin α5	38	56
GAPDH	38.4	39.5
Ratio	1	1.4
Normalized ratio	1	1.4

Figure 3 (d)

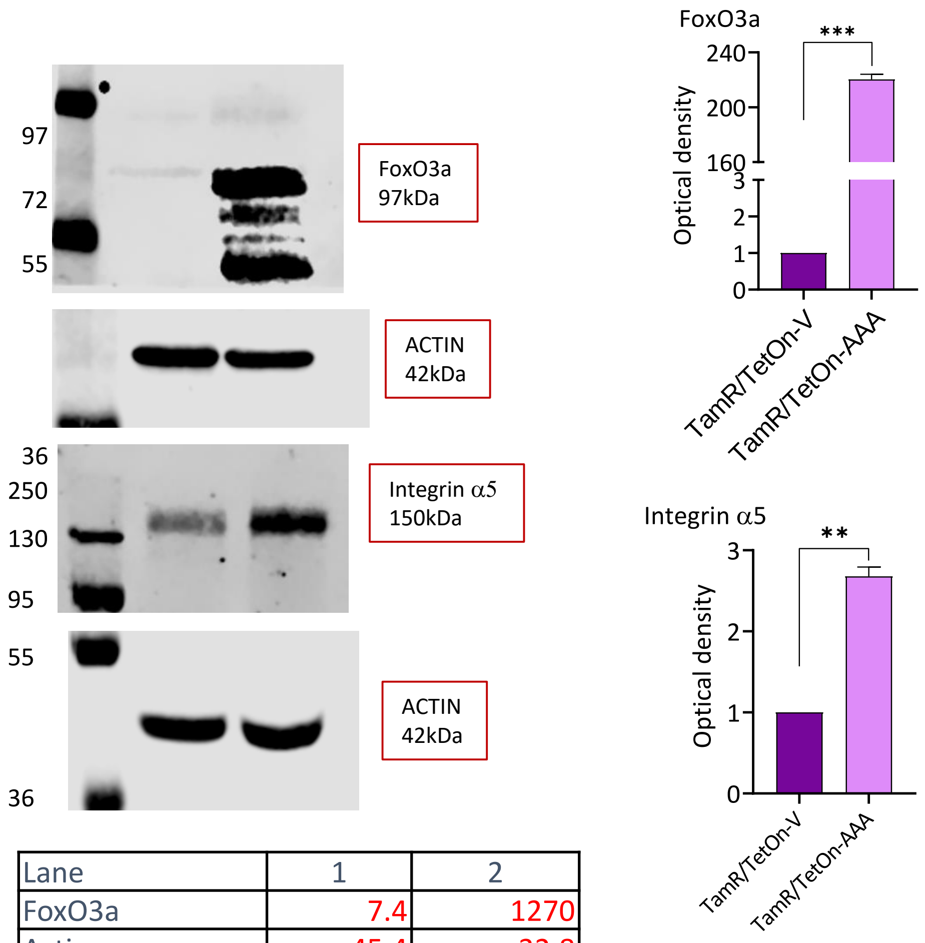

Lane	1	2
FoxO3a	7.4	1270
Actin	45.4	32.8
Ratio	0.2	37.6
Normalized ratio	1	196

Lane	1	2
Integrin α5	5.5	15.4
Actin	30.9	31.2
Ratio	0.2	0.5
Normalized ratio	1	2.5

Figure 3 (e)

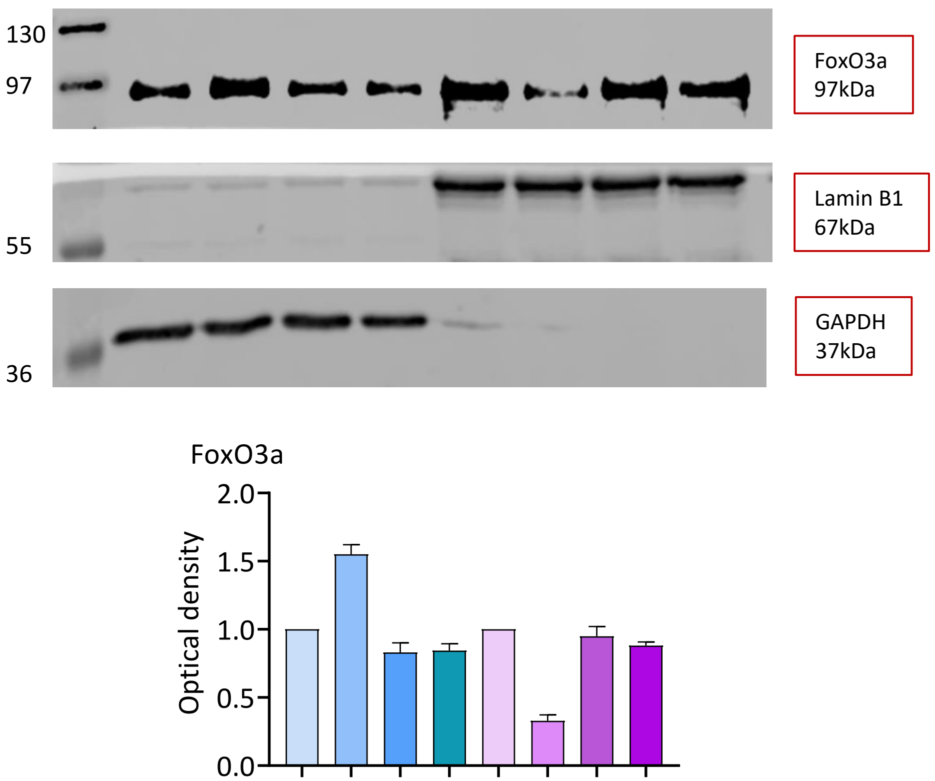

Lane	1	2	3	4	5	6	7	8
FoxO3a	6.5	9.4	5.6	4.4	9.9	3.3	9.7	9
Lamin B1					6.8	7.2	7.1	7.6
GAPDH	8.4	7.6	7.6	6.4				
Ratio	0.8	1.2	0.7	0.7	1.4	0.5	1.4	1.2
Normalized ratio	1	1.5	0.88	0.88	1	0.36	1	0.86

Figure 3 (f)

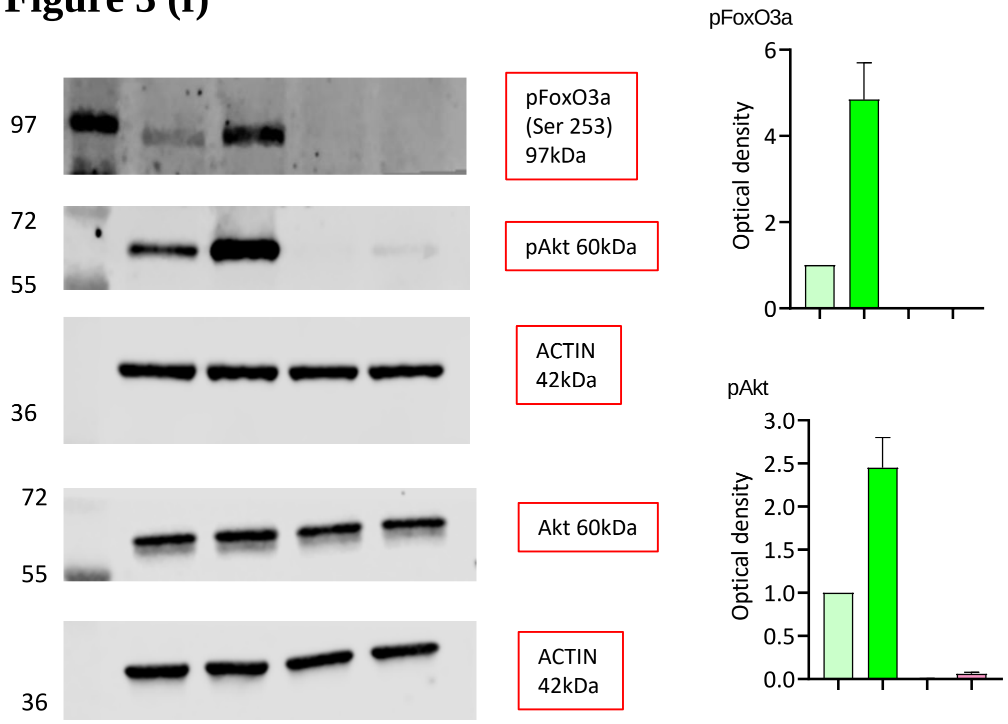

Lane	1	2	3	4
pFoxO3a	1.7	7.5	0	0
Actin	6.2	6.1	5.7	5.4
Ratio	0.3	1.2	0	0
Normalized ratio	1	4	0	0

Lane	1	2	3	4
pAkt	4.3	9.2	0	0.18
Actin	6.2	6.1	5.7	5.4
Ratio	0.7	1.5	0	0.03
Normalized ratio	1	2.1	0	0.04

Lane	1	2	3	4
Akt	7.4	7.8	6.9	7
Actin	14.4	13.8	13.7	12.9
Ratio	0.5	0.6	0.5	0.5
Normalized ratio	1	1.2	1	1

Figure 3 (g)

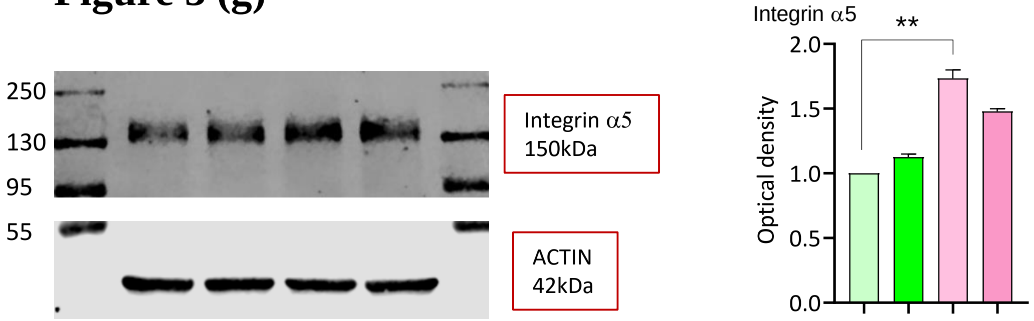

Lane	1	2	3	4
Integrin α5	6.36	6.8	9.2	8.5
Actin	6.6	6.2	5.7	5.9
Ratio	0.96	1.1	1.6	1.4
Normalized ratio	1	1.15	1.67	1.46

Figure 3 (h)

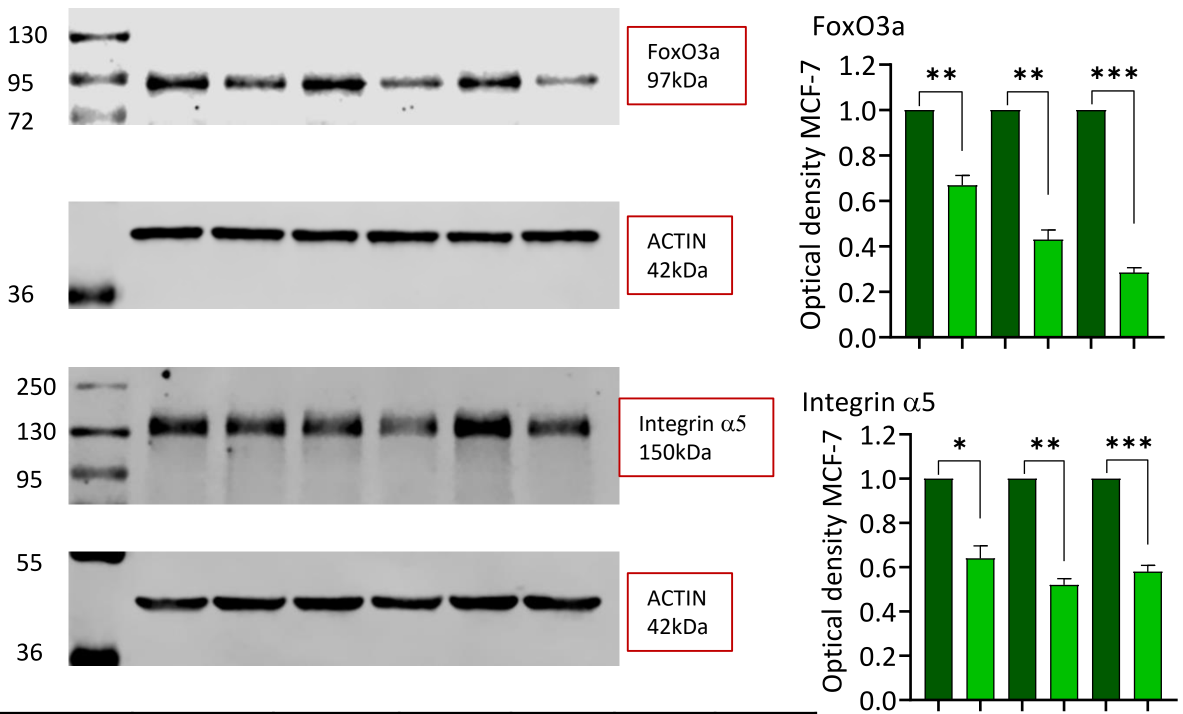

Lane	1	2	3	4	5	6
FoxO3a	8.8	5.3	9.3	4	7.2	2.7
Actin	30.1	27.8	21.4	23.7	18.3	26
Ratio	0.3	0.2	0.4	0.2	0.4	0.1
Normalized ratio	1	0.67	1	0.5	1	0.25

Lane	1	2	3	4	5	6
Integrin α5	15	13	13	8	23	11
Actin	9.4	13.1	14.3	13.8	16.1	14.3
Ratio	1.6	1	0.9	0.58	1.4	0.8
Normalized ratio	1	0.63	1	0.6	1	0.6

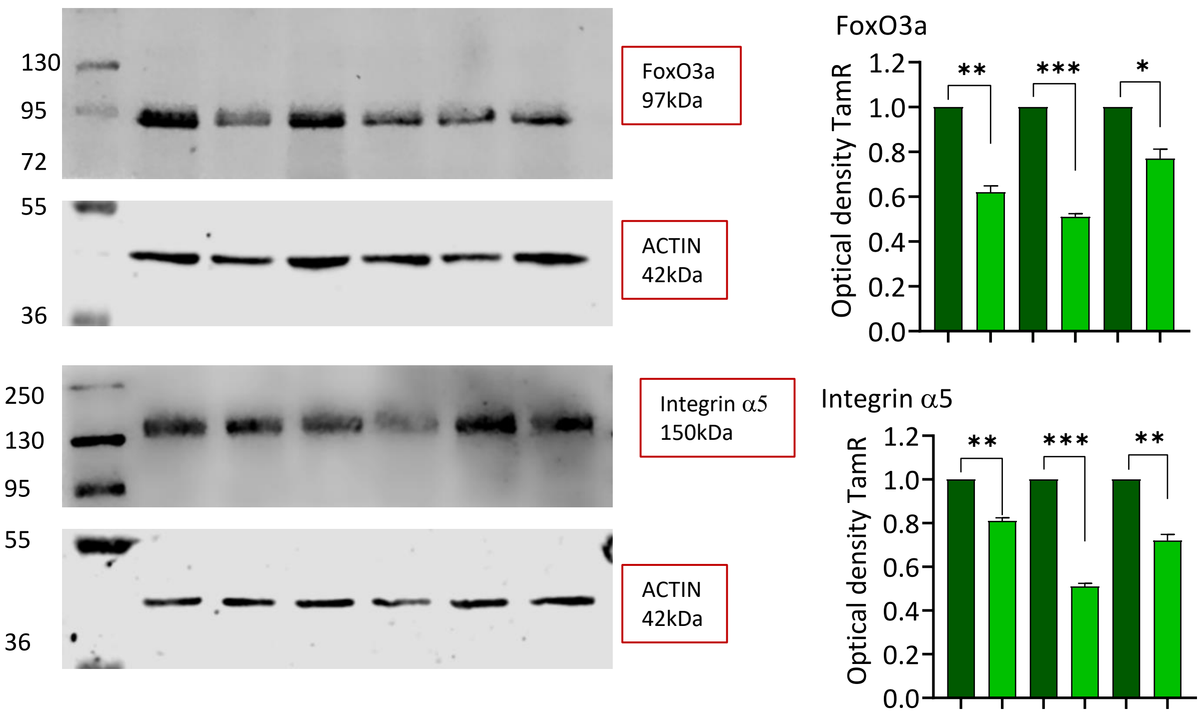

Lane	1	2	3	4	5	6
FoxO3a	9	3	7	3.5	3	4
Actin	13.5	8.10	14	13.4	6.6	14.4
Ratio	0.7	0.4	0.5	0.26	0.4	0.3
Normalized raio	1	0.6	1	0.5	1	0.75

Lane	1	2	3	4	5	6
Integrin α5	3	2.8	3	1.3	3.3	3.2
Actin	9.92	11.3	9.5	8.5	12.4	14.6
Ratio	0.3	0.25	0.3	0.15	0.3	0.2
Normalized ratio	1	0.8	1	0.5	1	0.7

Figure 4 (b)

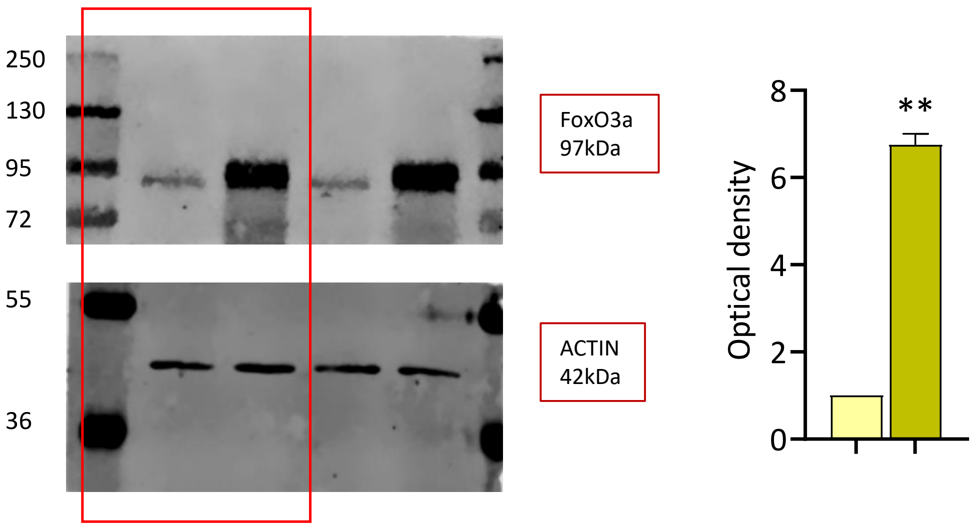

Lane	1	2
FoxO3a	1.5	14.2
Actin	2.67	3.63
Ratio	0.6	3.9
Normalized ratio	1	6.5

Figure 4 (c)

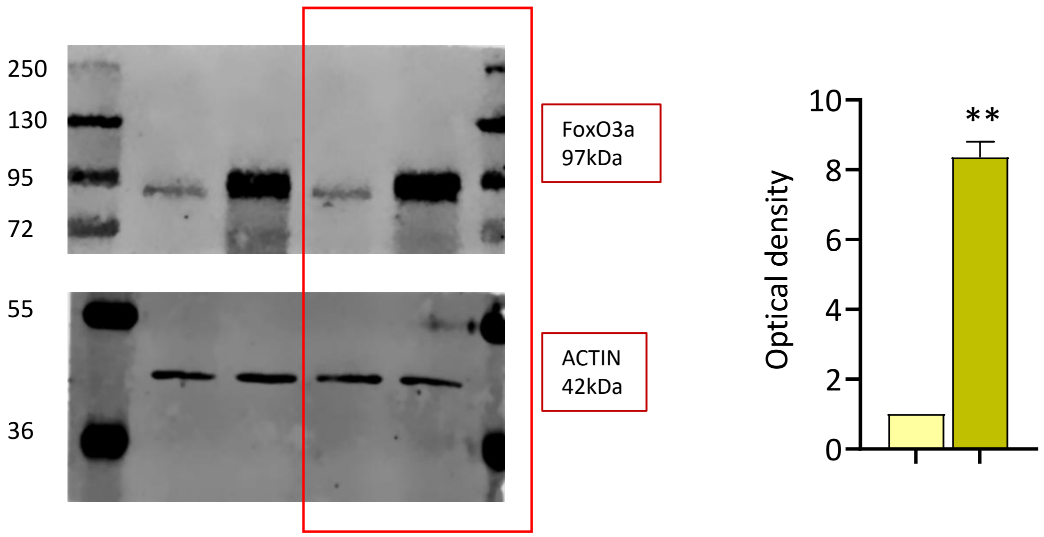

Lane	1	2
FoxO3a	1.6	15.1
Actin	2.79	2.87
Ratio	0.6	5.3
Normalized ratio	1	8.8

Figure 4 (e)

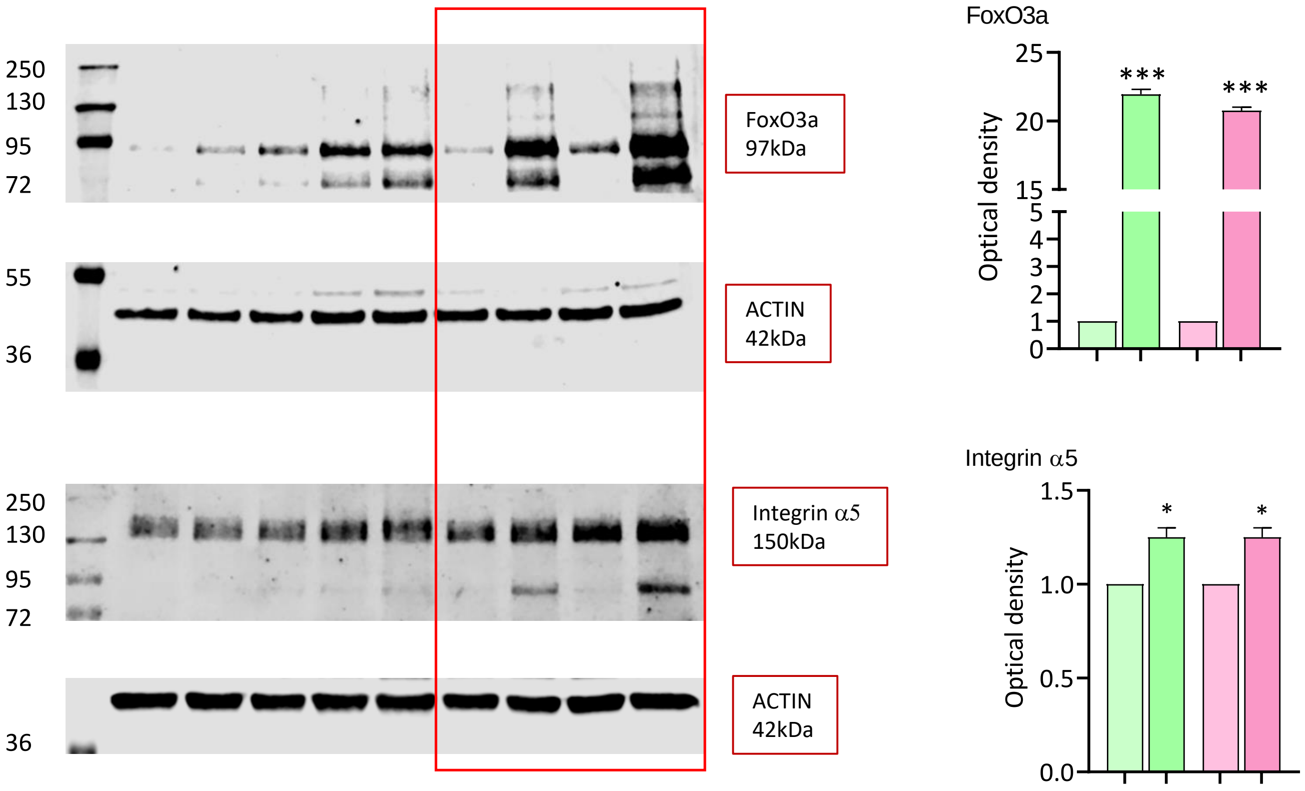

Lane	1	2	3	4
FoxO3a	7.5	171	23.2	521
Actin	23.8	25.6	30.7	31.8
Ratio	0.3	6.7	0.8	16.4
Normalized ratio	1	22.3	1	20.5

Lane	1	2	3	4
Integrin α5	1	1.4	1.7	2.3
Actin	30.8	33.8	51.9	59.5
Ratio	0.03	0.04	0.03	0.04
Normalized ratio	1	1.33	1	1.33

Figure 5 (a)

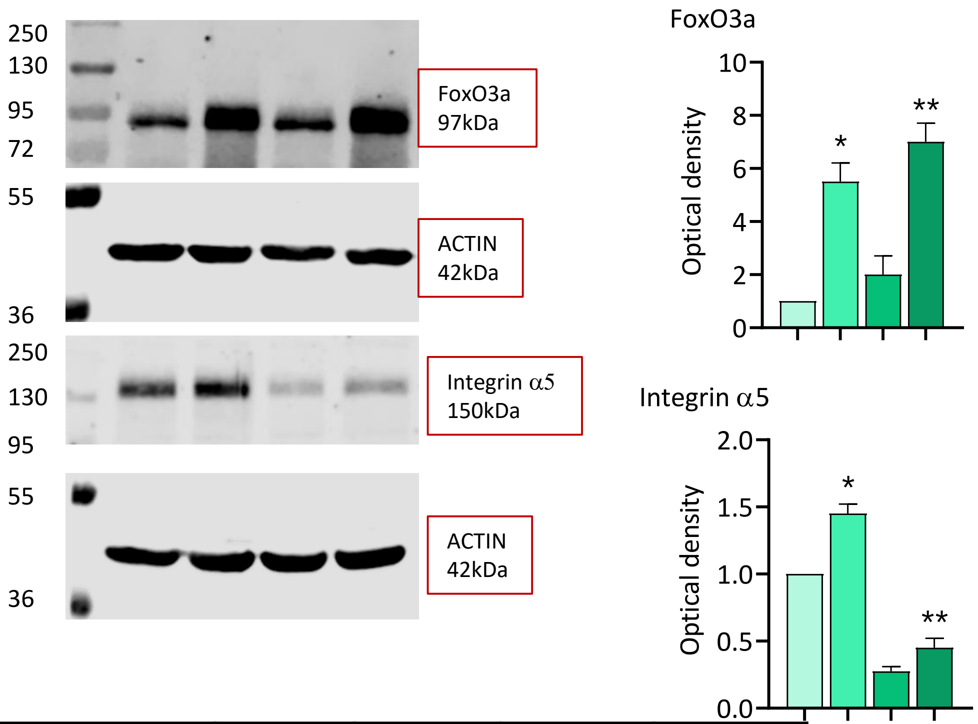

Lane	1	2	3	4
FoxO3a	2	8.5	2.9	11.8
Actin	57	45.1	30.1	37.3
Ratio	0.04	0.2	0.1	0.3
Normalized ratio	1	5	2.5	7.5

Lane	1	2	3	4
Integrin α5	5.2	6.9	1.4	2.5
Actin	54.7	54.4	70.7	75.2
Ratio	0.09	0.13	0.02	0.033
Normalized ratio	1	1.44	0.2	0.4

Figure 5 (b)

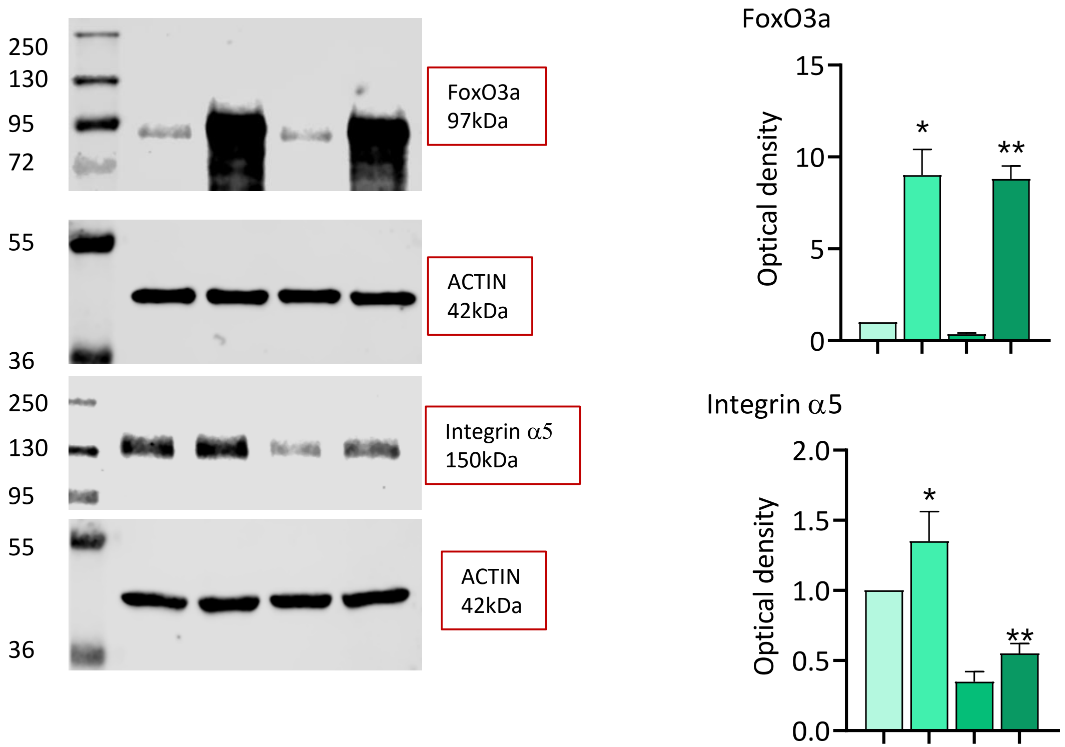

Lane	1	2	3	4
FoxO3a	3.7	32.1	3.3	21.4
Actin	61.6	58.1	60.4	43.9
Ratio	0.06	0.6	0.05	0.5
Normalized ratio	1	10	0.83	8.3

Lane	1	2	3	4
Integrin α5	1	1.3	0.4	0.64
Actin	46.7	45.9	47.7	58
Ratio	0.02	0.03	0.008	0.01
Normalized ratio	1	1.5	0.4	0.5

Appendix A (f)

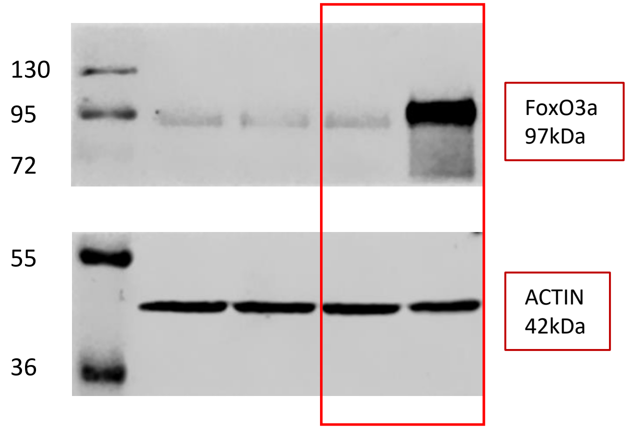

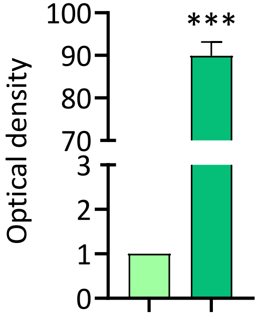

Lane	1	2
FoxO3a	1	60
Actin	29.9	23.1
Ratio	0.03	2.6
Normalized ratio	1	87

Appendix A (i)

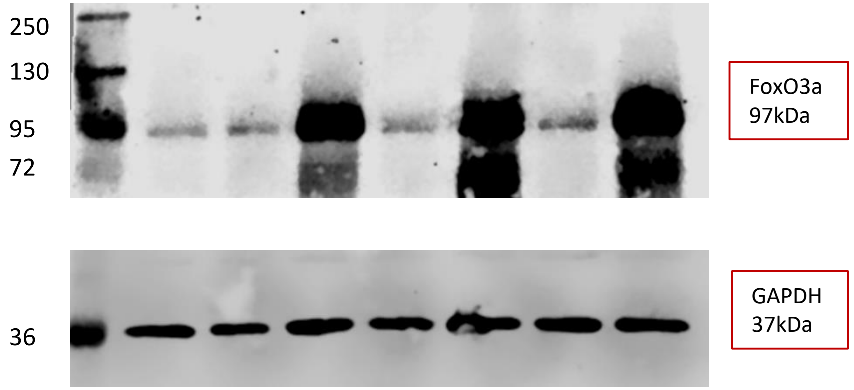

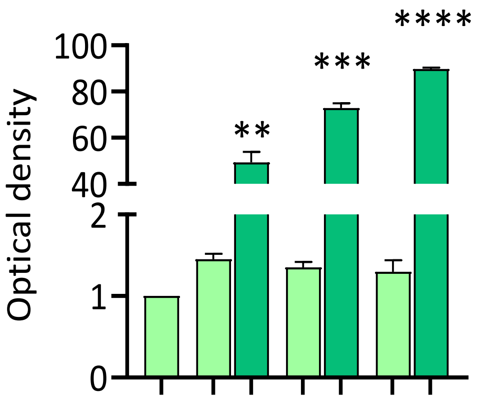

Lane	1	2	3	4	5	6	7
FoxO3a	1.1	1.1	74	1.2	82.2	1.8	100
GAPDH	8.51	7.40	14.3	7	10.2	13.1	10.6
Ratio	0.1	0.15	5.2	0.17	8.1	0.14	11.5
Normalized ratio	1	1.5	52	1.7	81	1.4	94

Appendix B (a)

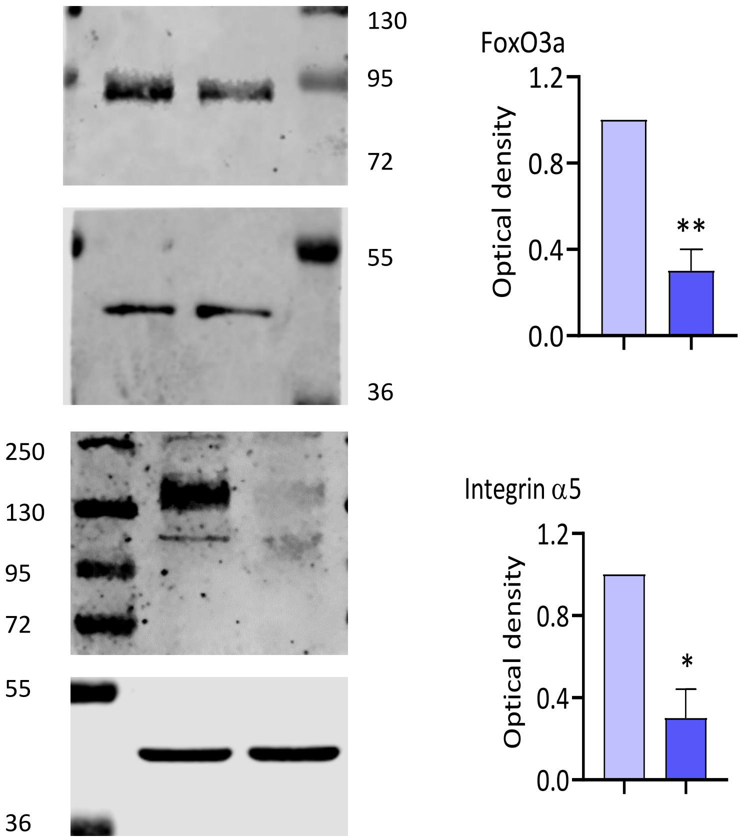

Lane	1	2
FoxO3a	6	3
Actin	9.2	9.2
Ratio	0.65	0.4
Normalized ratio	1	0.46

Lane	1	2
Integrin α5	8.5	1.4
Actin	49.9	53.7
Ratio	0.2	0.03
Normalized ratio	1	0.2

Appendix B (b)

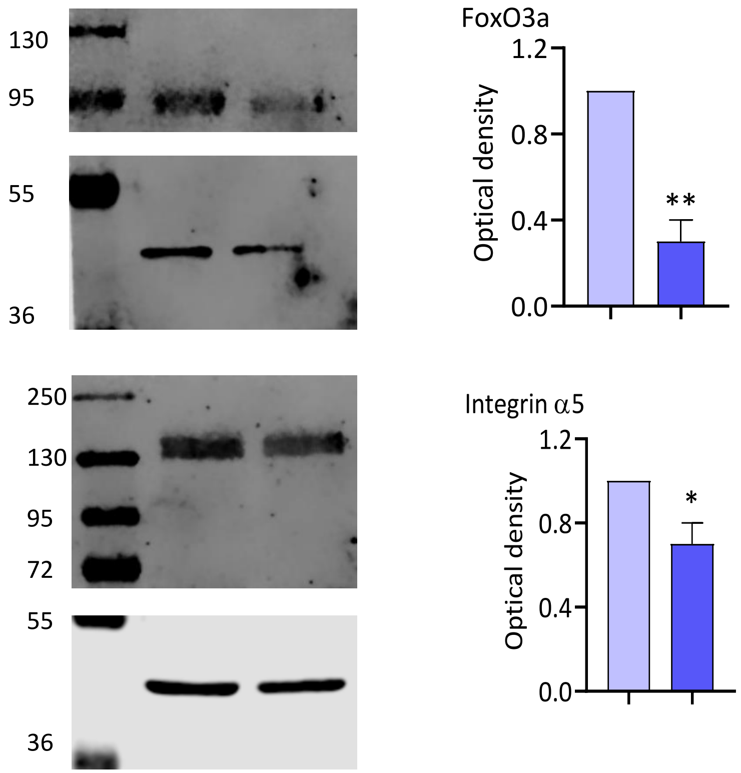

Lane	1	2
FoxO3a	2.2	0.67
Actin	1.7	1.3
Ratio	1.3	0.5
Normalized ratio	1	0.38

Lane	1	2
Integrin α5	4.8	3.9
Actin	46.8	44.2
Ratio	0.1	0.08
Normalized ratio	1	0.8

Appendix B (c)

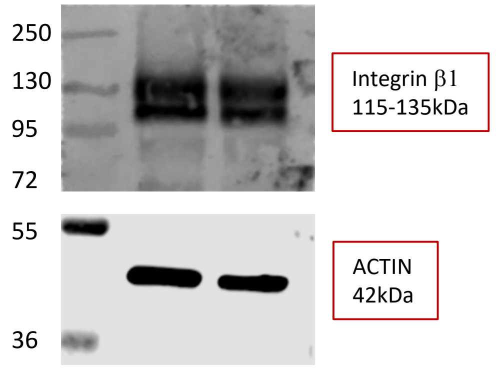

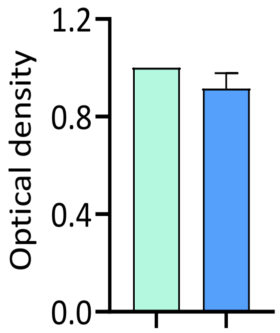

Lane	1	2
Integrin β1	25	18.5
Actin	98.5	74.7
Ratio	0.25	0.25
Normalized ratio	1	1

Appendix B (e)

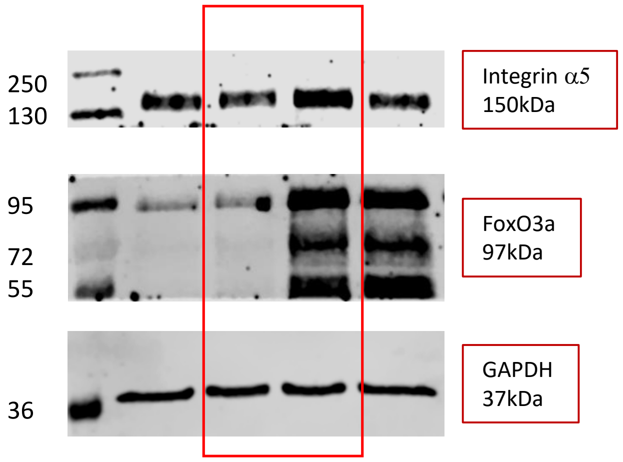

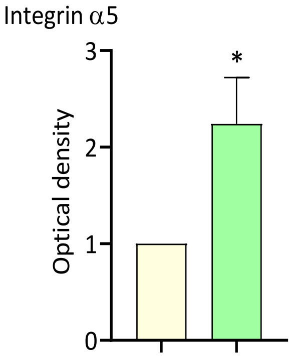

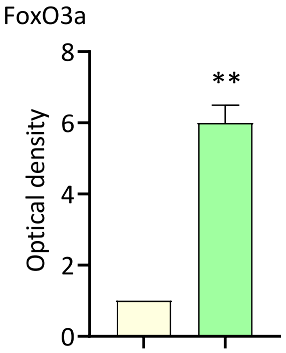

Lane	1	2
Integrin α5	11.2	24.3
GAPDH	11.3	12.2
Ratio	1	2
Normalized ratio	1	2

Lane	1	2
FoxO3a	2.4	16
GAPDH	11.3	12.2
Ratio	0.2	1.3
Normalized ratio	1	6.5

Appendix B (f)

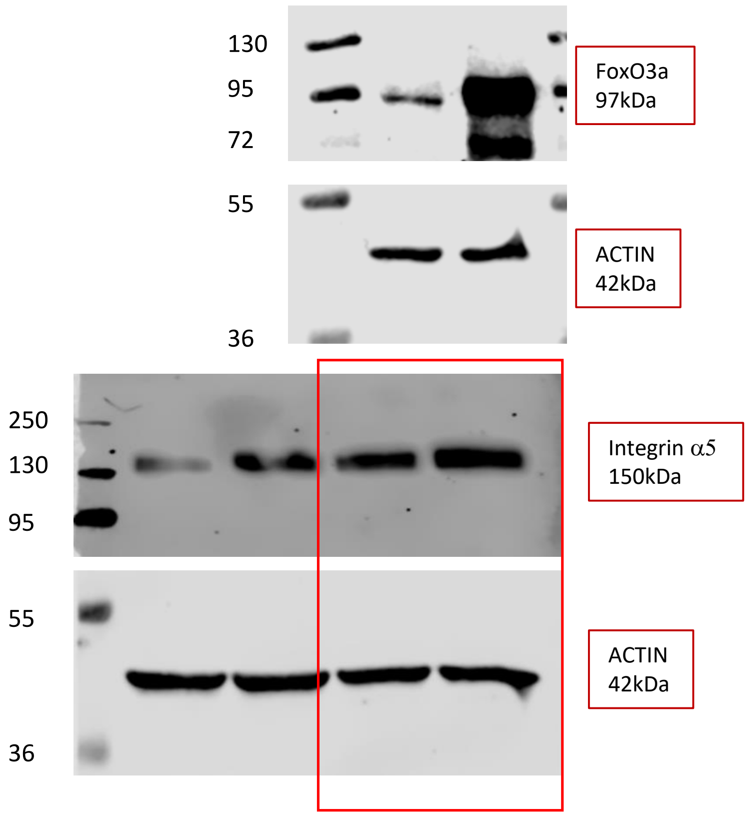

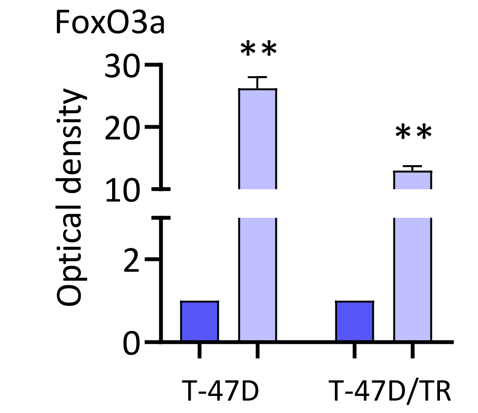

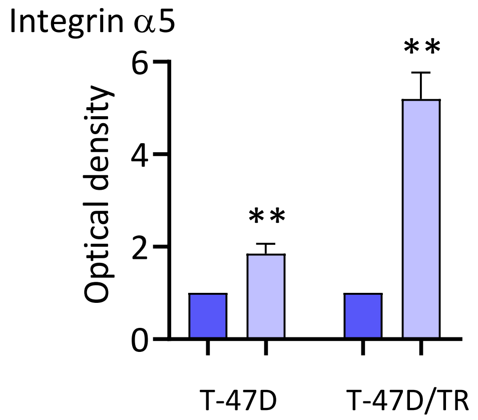

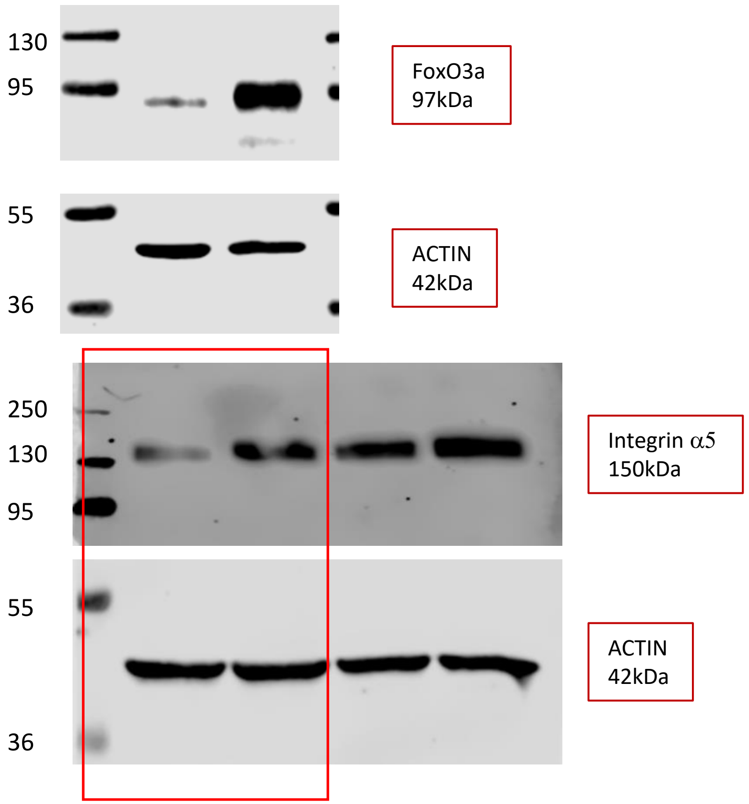

Lane	1	2
FoxO3a	4.8	183
Actin	29.1	33.4
Ratio	0.2	5.5
Normalized ratio	1	27.5

Lane	1	2
Integrin α5	1.2	2.3
Actin	39.9	47
Ratio	0.03	0.05
Normalized ratio	1	1.7

Lane	1	2
FoxO3a	1.3	9.9
Actin	29.1	18.9
Ratio	0.04	0.5
Normalized ratio	1	12.5

Lane	1	2
Integrin α5	2.8	11.7
Actin	51.7	45.2
Ratio	0.05	0.3
Normalized ratio	1	6

Appendix B (g)

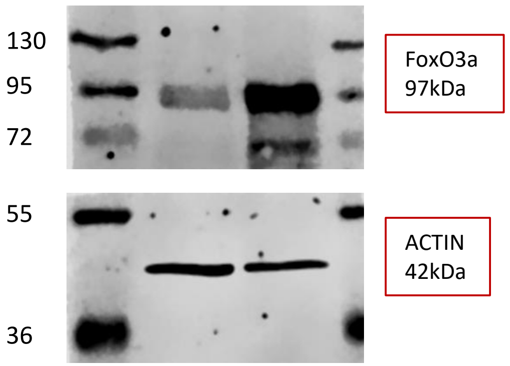

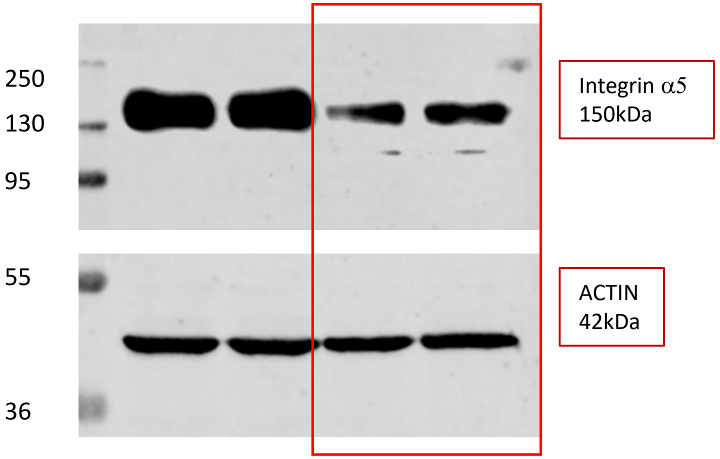

Lane	1	2
FoxO3a	4	50
Actin	34.2	21.1
Ratio	0.1	2.4
Normalized ratio	1	24

Lane	1	2
Integrin α5	2	2.8
Actin	55.4	60.8
Ratio	0.04	0.05
Normalized ratio	1	1.2

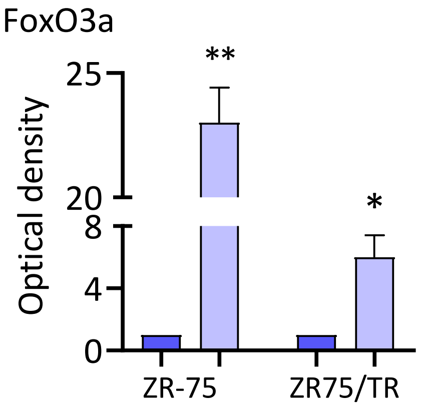

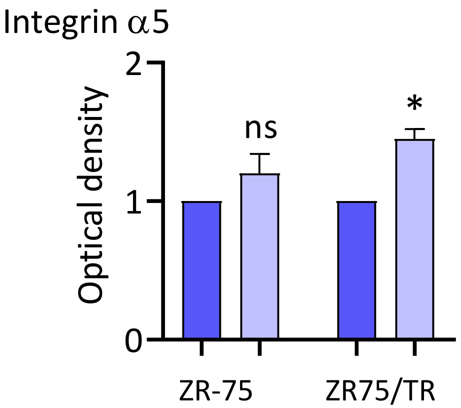

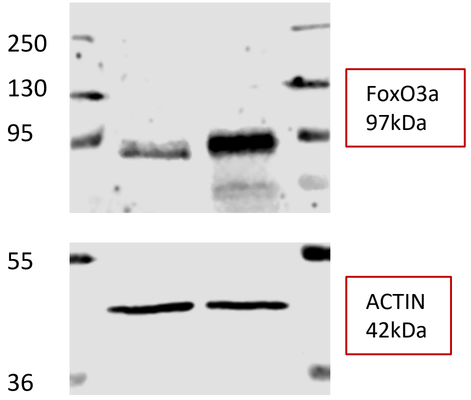

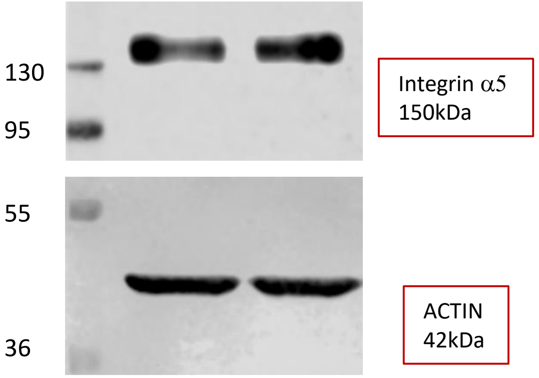

Lane	1	2
FoxO3a	5	21
Actin	24.3	21.7
Ratio	0.2	1
Normalized ratio	1	5

Lane	1	2
Integrin α5	1.7	2
Actin	73.8	59.5
Ratio	0.02	0.03
Normalized ratio	1	1.5

Appendix B (l)

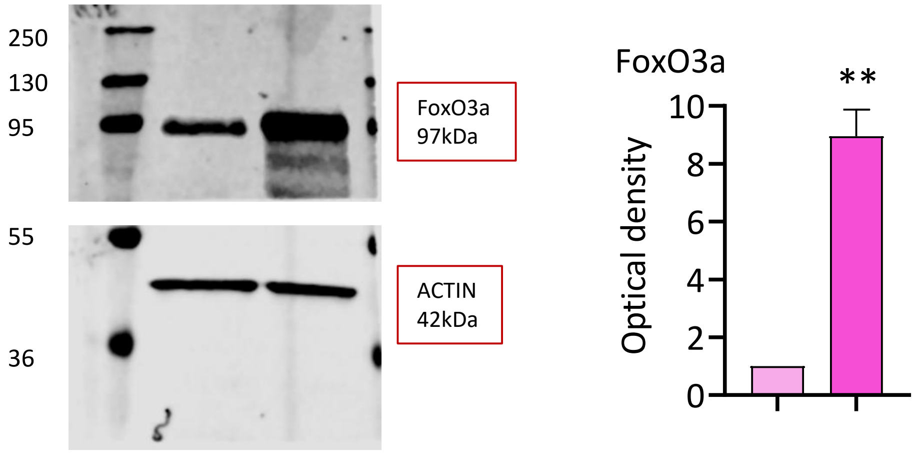

Lane	1	2
FoxO3a	17.1	166
Actin	25.1	25.4
Ratio	0.7	6.5
Normalized ratio	1	9.3

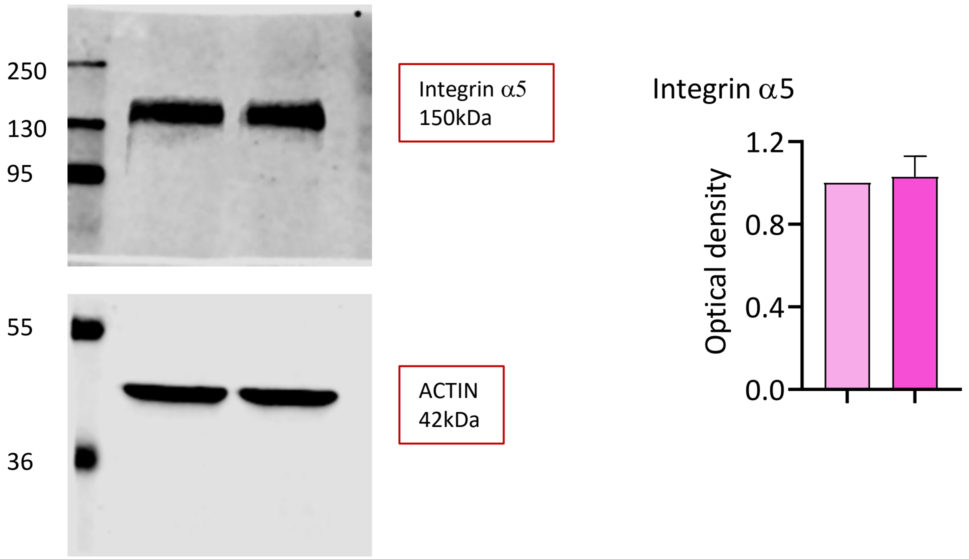

Lane	1	2
Integrin α5	32.2	35.7
Actin	49.8	49.2
Ratio	0.65	0.72
Normalized ratio	1	1.1

Appendix C (a)

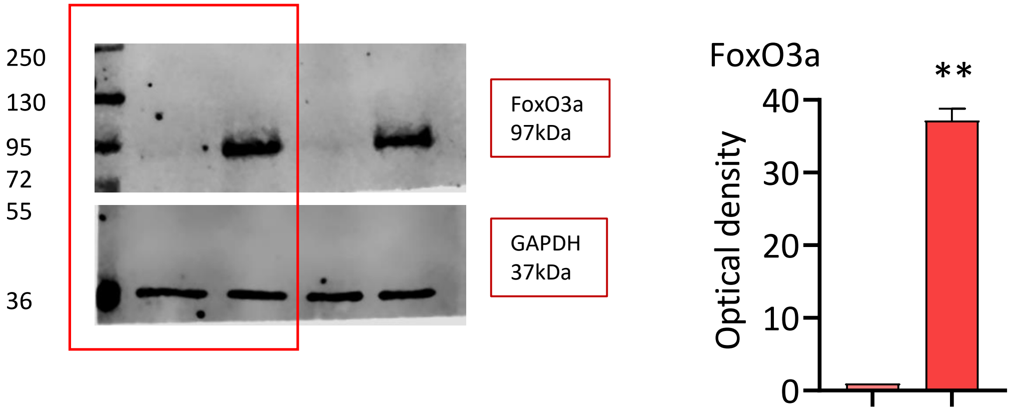

Lane	1	2
FoxO3a	0.2	7
GAPDH	3.1	3.04
Ratio	0.06	2.3
Normalized ratio	1	38.3

Appendix C (b)

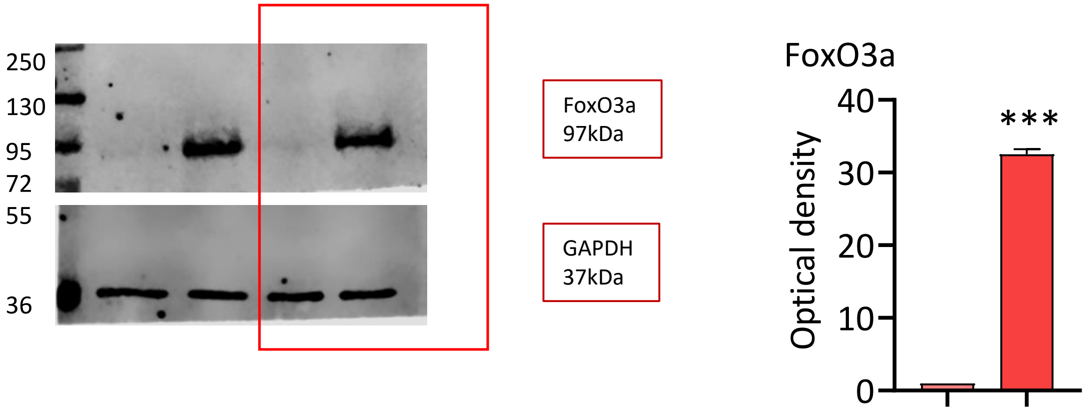

Lane	1	2
FoxO3a	0.2	6.2
GAPDH	3.8	3.8
Ratio	0.05	1.6
Normalized ratio	1	32
